# Gibberellins Inhibit Nodule Senescence and Stimulate Nodule Meristem Bifurcation in Pea (*Pisum sativum* L.)

**DOI:** 10.3389/fpls.2019.00285

**Published:** 2019-03-15

**Authors:** Tatiana A. Serova, Anna V. Tsyganova, Igor A. Tikhonovich, Viktor E. Tsyganov

**Affiliations:** ^1^Laboratory of Molecular and Cellular Biology, Department of Biotechnology, All-Russia Research Institute for Agricultural Microbiology, Russian Academy of Agricultural Sciences, Saint Petersburg, Russia; ^2^Department of Genetics and Biotechnology, Saint Petersburg State University, Saint Petersburg, Russia

**Keywords:** *Rhizobium–*legume symbiosis, nodule senescence, cysteine protease, ethylene, abscisic acid, gibberellins, meristem

## Abstract

The development of nitrogen-fixing nodules formed during *Rhizobium*–legume symbiosis is strongly controlled by phytohormones. In this study, we investigated the effect of gibberellins (GAs) on senescence of pea (*Pisum sativum*) symbiotic nodules. Pea wild-type line SGE, as well as corresponding mutant lines SGEFix^-^-1 (*sym40*), SGEFix^-^-2 (*sym33*), SGEFix^-^-3 (*sym26*), and SGEFix^-^-7 (*sym27*), blocked at different stages of nodule development, were used in the study. An increase in expression of the *GA2ox1* gene, encoding an enzyme involved in GA deactivation (GA 2-oxidase), and a decrease in the transcript abundance of the *GA20ox1* gene, encoding one of the enzymes involved in GA biosynthesis (GA 20-oxidase), were observed in analyzed genotypes during nodule aging. A reduction in the amount of bioactive GA_3_ was demonstrated by immunolocalization in the early senescent mutant and wild-type lines during aging of symbiotic nodules. Down-regulated expression of senescence-associated genes encoding cysteine proteases 1 and 15a, thiol protease, bZIP transcription factor, 1-aminocyclopropane-1-carboxylate (ACC) synthase, ACC oxidase, and aldehyde oxidase was observed in the nodules of wild-type plants treated with exogenous GA_3_ relative to the untreated plants. GA_3_-treated plants also showed increases in nodule size and the nitrogen fixation zone, and decreases in the number of nodules and the senescence zone. Immunogold localization revealed higher levels of GA_3_ in the peribacteroid spaces in symbiosomes than in the matrix of infection threads. Furthermore, a decrease in GA_3_ label in mature and senescent symbiosomes in comparison with juvenile symbiosomes was observed. These results suggest a negative effect of GAs on the senescence of the pea symbiotic nodule and possible involvement of GAs in functioning of the mature nodule. Simultaneously, GA_3_ treatment led to nodule meristem bifurcation, indicating a possible role of GAs in nodule meristem functioning.

## Introduction

Legume–*Rhizobium* interactions culminate in the formation of nitrogen-fixing nodules. Rhizobia growing near the plant adhere to the root hair and eventually penetrate the root, initiating the formation of an infection thread (Brewin, 2004; Tsyganova and Tsyganov, 2017). When the infection thread reaches reactivated cortical root cells, which form a nodule primordium, rhizobia are released into the host cell cytoplasm from unwalled infection droplets (Brewin, 2004). After release, rhizobia differentiate into bacteroids and become surrounded by a peribacteroid membrane; these form symbiosomes, organelle-like structures in which bacteroids fix nitrogen (Tsyganova et al., 2018).

If the nodule meristem functions for a long time, nodules of an indeterminate type are formed with different histological zones, which include the meristem (zone I), the infection zone (zone II), the nitrogen fixation zone (zone III), and the senescence zone (zone IV) (Guinel, 2009).

Senescence completes symbiotic nodule development and is accompanied by the destruction of symbiotic partners, large-scale protein degradation, and remobilization of nutrients to other plant organs (Puppo et al., 2005; Serova and Tsyganov, 2014). In particular, the catabolism of leghemoglobin, which is one of the most abundant proteins in the nodule, is observed during nodule senescence resulting in a color change of aged nodules from pink to green. Senescence in the indeterminate nodule is associated with the senescence zone formed at the base of the nodule and spreads toward its apical part and periphery (Pérez Guerra et al., 2010; Dupont et al., 2012). Hormonal regulation has a major impact on symbiotic nodule development (Ferguson and Mathesius, 2014; Tsyganova and Tsyganov, 2015, 2018). Current data suggest that both ethylene and abscisic acid (ABA) contribute to the aging of the symbiotic nodule (Puppo et al., 2005; Van de Velde et al., 2006; Karmarkar, 2014; Serova et al., 2017). In contrast, based on expression analysis of the nodules of *Medicago truncatula* (Van de Velde et al., 2006) and pea (Serova et al., 2017), it has been suggested that gibberellins (GAs) may have a negative impact on nodule senescence.

Gibberellins are a large group of diterpenoid carboxylic acids in higher plants. GAs stimulate organ growth, causing the enhancement of cell elongation and cell division (Hedden and Thomas, 2012). GA biosynthesis includes several steps catalyzed by terpene cyclases (Hedden and Thomas, 2012). The first steps involve the production of GA_12_, the common precursor of all types of GAs in plants (Hedden and Phillips, 2000). GA_12_ can be converted to another GA precursor, GA_53_. The final stages of GA biosynthesis are catalyzed by GA 20-oxidase and GA 3-oxidase. Their activity contributes to the content of bioactive forms of GA in the plant. In pea, GA 20-oxidases encoded by *PsGA20ox* genes (*PsGA20ox1*, *PsGA20ox2*) are involved in different stages of GA biosynthesis. They mainly catalyze the conversion of GA_12_ to GA_9_ and GA_53_ to GA_20_, where GA_9_ and GA_20_ are precursors of bioactive GAs (García-Martínez et al., 1997). The conversion of GA_9_ and GA_20_ to bioactive forms of GAs is catalyzed by GA 3-oxidases (García-Martínez et al., 1997; Hedden and Thomas, 2012).

GA_1_, GA_3_, GA_4_, GA_5_, and GA_7_ are the most common biologically active forms in higher plants (Hayashi et al., 2014). Along with bioactive forms, plants also contain inactive forms of GAs, including the precursors and metabolites of active GAs. Inactive GAs are present at higher concentrations and may perform yet unknown functions (Hedden and Thomas, 2012; Hayashi et al., 2014). There are several mechanisms of GA inactivation, the most prevalent of which is 2β-hydroxylation, catalyzed by GA 2-oxidases (GA2oxs) (Thomas et al., 1999; Hedden and Thomas, 2012). In pea, conversion of bioactive GAs, GA_1_ and GA_4_, and of its precursors GA_9_ and GA_20_, to inactive catabolites occurred by C_19_-GA 2-oxidases encoded by the *PsGA2ox1* and *PsGA2ox2* genes (Lester et al., 1999; Martin et al., 1999; Hedden and Thomas, 2012). Inactivation of the precursors GA_12_ and GA_53_ is catalyzed by C_20_-GA 2-oxidases (Hedden and Thomas, 2012).

Optimal GA levels differ during various stages of plant development and are maintained through feed-back and feed-forward regulation of GA metabolism (Weston et al., 2008; Hedden and Thomas, 2012). Bioactive GAs reduce GA biosynthesis and enhance GA deactivation (Weston et al., 2008). A GA signal transduction pathway is triggered by the binding of GAs to the soluble receptor GID1 (GIBBERELLIN-INSENSITIVE DWARF 1) (Ueguchi-Tanaka et al., 2005). Downstream signal transduction pathways involve DELLA proteins, which are key repressors of GA responses (Davière and Achard, 2013). After GA binding, the formation of a GA-GID1-DELLA complex occurs with its subsequent degradation by the 26S proteasome in the nucleus (Sun, 2011).

During nodulation, GAs are involved in the negative control of rhizobial infection and the positive regulation of nodule development (Lievens et al., 2005; Maekawa et al., 2009; Ferguson et al., 2011; Hayashi et al., 2014; McAdam et al., 2018). Up-regulation of GA biosynthetic genes, *GA20ox* and *GA3ox*, was observed in the early stages of nodulation in *Sesbania rostrata* (Lievens et al., 2005), *Glycine max* (Hayashi et al., 2012), and *Lotus japonicus* (Kouchi et al., 2004). The negative effect of GAs on rhizobial infection in *L. japonicus* and *S. rostrata* was accompanied by suppressed expression of the transcription factors NIN (Nodule Inception) and NSP (Nodulation Signaling Pathway) (Kouchi et al., 2004; Lievens et al., 2005; Maekawa et al., 2009). Analysis of the *M. truncatula* root transcriptome revealed that Nod factor perception led to spatial-temporal activation of genes involved in GA biosynthesis and catabolism (Larrainzar et al., 2015). Within the first hours of infection, an increase in the expression of GA deactivation genes was shown, which may promote the Nod factor signaling pathway (Larrainzar et al., 2015). However, up-regulation of GA biosynthesis genes was later observed, which likely causes an increase in GA content and may limit further infection (Larrainzar et al., 2015). The negative impact of GAs on rhizobial infection is mediated through destruction of DELLA proteins. It was previously shown that DELLA proteins interact with and activate a wide set of transcription factors involved in Nod factor signaling [NSP1, NSP2, IPD3 (interacting protein with DMI3), NF-YA1 (nuclear factor-YA1), and ERN1 (ERF required for nodulation 1)] (Fonouni-Farde et al., 2016; Jin et al., 2016). Furthermore, the positive effect of DELLA proteins was confirmed by poor nodulation of *della* mutants in *M. truncatula* (Jin et al., 2016).

There is evidence to suggest that different optimal levels of bioactive GAs are required at different stages of nodulation (Ferguson et al., 2005, 2011). Moreover, optimal levels seem to be species-specific and to depend on growth conditions. Inhibition of GA biosynthesis reduced nodulation in *S. rostrata* (Lievens et al., 2005). In pea, reduced GA levels inhibited nodulation, as demonstrated using a series of GA biosynthesis mutants, including the severely GA-deficient *na-1* pea mutant (Ferguson et al., 2005). In *L. japonicus*, treatment of wild-type plants with exogenous GA_3_ induced the initiation of divisions in the pericycle leading to formation of pseudo-nodules (Kawaguchi et al., 1996). However, in the *snf1* and *snf2* mutants of *L. japonicus*, application of exogenous GA_3_ suppressed spontaneous nodulation by inhibiting the NSP (Maekawa et al., 2009). High concentrations of exogenous GA_3_ inhibited root hair infection in *S. rostrata* (10^-5^ M) (Lievens et al., 2005) and *L. japonicus* (10^-6^, 10^-7^ M) (Maekawa et al., 2009), and also reduced nodulation in pea (10^-3^ M) (Ferguson et al., 2005) and *M. truncatula* (10^-7^–10^-4^ M) (Fonouni-Farde et al., 2016; Jin et al., 2016). In contrast, low concentrations (10^-9^ M) of exogenous GA_3_ promoted nodulation in pea plants (Ferguson et al., 2005). Further overexpression of a GA signaling component, *SLEEPY1*, resulted in fewer nodules in transgenic roots of *L. japonicus* than in wild-type roots (Maekawa et al., 2009). However, the GA_1_-overproducing *sln* mutant of pea, which is strongly blocked in GA deactivation, formed nodules only on lateral roots and had the same number of nodules as the wild-type (Ross et al., 1995; Lester et al., 1999; Ferguson et al., 2005, 2011).

An ambivalent role for GAs in nodulation was clarified in a recent study (McAdam et al., 2018). The authors confirmed the well-known negative effect of GAs on development of infection threads and demonstrated the positive effect on nodule tissue development and functioning of nitrogen-fixing nodules. The action of GAs was shown to be mediated by DELLA proteins, which, on the contrary, promote infection thread growth and inhibit the initiation of cortex cell division and nodule development (McAdam et al., 2018).

Little is known about the interactions between GAs and other hormones during nodulation. In an earlier study using the strongly GA-deficient *na-1* pea mutant, it was shown that low GA content resulted in elevated ethylene levels and decreased nodulation (Ferguson et al., 2011). This was confirmed by partial recovery of nodulation in the mutant *na-1* treated with an inhibitor of ethylene biosynthesis (Ferguson et al., 2011), while an increase in the level of GAs rescued the number and structure of mutant nodules (Ferguson et al., 2005). Recently, a detailed phenotypic characterization of the *na-1 ein2* double mutant and an analysis of epistatic interactions of the mutations revealed that, to some extent, GAs inhibit infection thread formation independently of ethylene, and that GAs facilitate the formation of nodules by partial inhibition of ethylene (McAdam et al., 2018).

Thus, a number of previous studies confirm involvement of GAs in the initial stages of *Rhizobium*–legume symbiosis. However, at present, the effect of GAs on the later stages of nodule development is not well understood. Therefore, the aim of this study was to identify a role for GAs in senescence of the symbiotic nodule of pea. It is known that in wild-type pea nodules, senescence is initiated at 4 weeks after inoculation (WAI) and actively developed in 6-week-old nodules (Kardailsky and Brewin, 1996; Serova et al., 2017, 2018). The study was carried out using pea wild-type line SGE and a number of mutant lines, SGEFix^-^-1 (*sym40*), SGEFix^-^-2 (*sym33*), SGEFix^-^-3 (*sym26*), and SGEFix^-^-7 (*sym27*), which are blocked at different stages of nodule development. Recently, we demonstrated the activation of nodule senescence in all analyzed mutants, which starts in 2-week-old nodules and is especially active in 4-week-old nodules (Serova et al., 2018). Based on data obtained from expression, immunolocalization, and pharmacological analyses, we suggest a negative effect of GAs on the senescence of the pea symbiotic nodule and possible involvement of GAs in the functioning of nodule meristem and cells from the nitrogen fixation zone.

## Materials and Methods

### Plant Material, Bacterial Strain, and Plant Growth Conditions

The pea (*Pisum sativum* L.) laboratory line SGE (Kosterin and Rozov, 1993) and corresponding mutant lines SGEFix^-^-1 (*sym40*), SGEFix^-^-2 (*sym33*), SGEFix^-^-3 (*sym26*), and SGEFix^-^-7 (*sym27*) were used in this study (Table 1). *Rhizobium leguminosarum* bv. *viciae* strain 3841 (Wang et al., 1982) was used as an inoculant. Methods for sterilization of seeds, plant inoculation, and growth conditions were described previously (Serova et al., 2017). For microscopic and expression analyses and quantitative measurement of nodulation, nodules were collected at 2, 4, and 6 WAI.

**Table 1:** Plant material used in the study.

Genotype	Mutant gene	Nodule phenotype	Reference
SGE		Wild type	Kosterin and Rozov, 1993; Tsyganov et al., 1998
SGEFix^-^-1	*sym40^∗^*	Hypertrophic infection threads and infection droplets; abnormal bacteroids	Tsyganov et al., 1994, 1998
SGEFix^-^-2	*sym33^∗∗^*	“Locked” infection threads, no bacterial release; in some cells and nodules infection droplets are formed and bacterial release occurs	Tsyganov et al., 1994, 1998; Voroshilova et al., 2001
SGEFix^-^-3	*sym26*^∗∗∗^	Premature degradation of symbiotic structures	Tsyganov et al., 2000; Serova et al., 2018
SGEFix^-^-7	*sym27*^∗∗∗^	Premature degradation of symbiotic structures	Tsyganov et al., 2013; Serova et al., 2018


*^∗^The *Sym40* gene is orthologous to the *Medicago truncatula EFD* gene (Nemankin, 2011). ^∗∗^The *Sym33* gene is orthologous to the *M. truncatula IPD3* gene (Ovchinnikova et al., 2011). ^∗∗∗^The gene sequence is unknown*.

### Expression Analysis of Genes Associated With GA Metabolism in SGE and Mutant Lines

Primers for one GA biosynthesis gene, *PsGA20ox1*, and one GA deactivation gene, *PsGA2ox1*, were designed previously (Weston et al., 2008; Serova et al., 2017). For RNA extraction, 2, 4, and 6 WAI nodules of SGE, mutants SGEFix^-^-1 (*sym40*), SGEFix^-^-2 (*sym33*), SGEFix^-^-3 (*sym26*), and SGEFix^-^-7 (*sym27*) were ground in liquid nitrogen. Total RNA from each sample was isolated using PureZol reagent (Bio-Rad, Hercules, CA, United States) according to the manufacturer’s recommendations. RNA quantity and purity were checked using a MultiNA electrophoresis system on microchips for analysis of nucleic acids (Shimadzu Corporation, Kyoto, Japan). Reverse transcription was performed on 1.5 μg total RNA treated with DNAse I (MBI Fermentas, Vilnius, Lithuania) using 200 U RevertAid Reverse Transcriptase and 0.5 μg Oligo(dT)_18_ (MBI Fermentas) for cDNA synthesis under manufacturer-recommended conditions. cDNA synthesis was carried out in 20 μl of reaction mix, and the resulting cDNAs were diluted five times for following use. The reaction was carried out in an automated C1000^TM^ Thermal Cycler (Bio-Rad).

For gene expression quantification, relative real-time PCR was performed in a C1000^TM^ Thermal Cycler combined with the optical module CFX96^TM^ Real-Time System (Bio-Rad), using iQ SYBR Green Supermix (Bio-Rad) according to manufacturer’s instructions. Results of reactions were processed using Bio-Rad CFX Manager software (Bio-Rad). Relative expression was calculated with the 2^-ΔΔC_T_^ method using the reference gene *PsGapC1* (accession number L07500.1). Two-WAI nodules of SGE were used as a calibrator for calculation of relative transcript abundance. For *PsGA20ox1* transcript abundance, 6-WAI nodules were used as a calibrator. Reactions were carried out in three technical replicates and averaged. Statistical treatment of experimental results was carried out with Microsoft Excel software. Statistically significant differences were calculated using one-way ANOVA at *P*-value ≤ 0.05. Experiments were performed in three replicates with six to eight plants per variant.

### GA_3_ Immunolabeling and Confocal Microscopy

Nodules of SGE and of the mutants SGEFix^-^-1 (*sym40*), SGEFix^-^-2 (*sym33*), SGEFix^-^-3 (*sym26*), and SGEFix^-^-7 (*sym27*) at 2, 4, and 6 WAI were fixed in freshly prepared 4% paraformaldehyde buffered in PBS (136 mM NaCl, 2.68 mM KCl, 10 mM Na_2_HPO_4_, 1.7 mM KH_2_PO_4_, pH 7.4) with addition of 3% *N*-ethyl-*N′*-(3-dimethylaminopropyl) carbodiimide hydrochloride and 0.1% Triton X-100 (Sigma-Aldrich, Dorset, United Kingdom). Samples were fixed under vacuum (-0.9 bar), for 7 min three times with 15 min intervals using a VacuuBrand ME 1C vacuum pump (Vacuubrand, Wertheim, Germany), and incubated overnight at 4°C. Nodules were rinsed with PBS three times, with 15 min intervals, and stained with 0.5% toluidine blue solution in PBS for 1 h. Washing of the residual dye was carried out with PBS two times, with 15 min intervals. Samples were subsequently molded in 3% agarose gel.

Sections (50 μm) were prepared at room temperature with a HM650V microtome (Microm, Walldorf, Germany). After this, GA_3_ immunolabeling and nuclei and bacteria staining were carried out in accordance with Serova et al. (2018). Anti-GA_3_ rat antibodies (Agrisera, Vännäs, Sweden) and goat anti-rat IgG Alexa Fluor 488 (Thermo Fisher Scientific, Waltham, MA, United States) were used as primary and secondary antibodies for GA_3_ immunolabeling, respectively. Sections were mounted in ProLong Gold antifade reagent (Thermo Fisher Scientific).

Control of GA_3_-specific signal was carried out using GA_3_-BSA conjugate (Agrisera) (Supplementary Figure S1). Anti-GA_3_ antibodies were incubated with GA_3_-BSA conjugate in a 1:40 ratio in PBS for 24 h at 4°C in a total volume of 100 μl. Then, the mix was used as primary antibodies for immunolabeling of pea nodules (Supplementary Figures S1A–C). Anti-GA_3_ antibodies were omitted as a control for specific binding of secondary antibodies in the absence of primary antibodies (Supplementary Figures S1D–F). Also, the control of specificity of GA_3_-antibodies in nuclei is shown (Supplementary Figures S1G–L).

Sections were analyzed using the laser scanning confocal system LSM 510 META (Carl Zeiss, Oberkochen, Germany) and ZEN2009 software (Carl Zeiss).

### Immunogold Labeling, Transmission Electron Microscopy, and Quantitative Analysis

Nodules of SGE and mutant SGEFix^-^-3 (*sym26*) at 2 WAI were used. Preparation of samples for transmission electron microscopy (TEM) and immunogold labeling of GA_3_ was done with ultrathin sections on gold grids as described by Tsyganova et al. (2009). A total of 15–20 nodules from at least five different plants were fixed in 2.5% glutaraldehyde in 0.06 M phosphate buffer (pH 7.2) at 4°C overnight. Samples were then rinsed in buffer and dehydrated in increasing concentrations of ethanol (30% at room temperature; 50%, 70%, 90%, and twice with 100% at 35°C) for 20 min at each step. Subsequently, specimens were gradually infiltrated with increasing concentrations of LR-White resin (Polysciences Europe, Eppelheim, Germany) at a ratio of 1:1, 1:2, and 1:3 mixed with ethanol (100%) at -20°C and finally embedded in LR-White resin and polymerized at -20°C for 48 h in small plastic containers using UV polymerization in a Leica EM AFS2 (Leica Microsystems, Wetzlar, Germany).

Ultrathin sections (90 nm) of the samples were obtained with a Leica EM UC7 ultramicrotome (Leica Microsystems) and blocked with a blocking solution (5% BSA, 0.5% goat serum, 0.05% cold water fish skin) and they were then washed in 0.1% acetylated BSA (BSA-C) in PBS. Sections were treated with the primary anti-GA_3_ rat antibodies (Agrisera) diluted 1:25 in 0.1% BSA-C in PBS at 4°C overnight. After four rinses in 0.1% BSA-C in PBS, samples were incubated with a 10 nm gold-conjugated secondary antibody goat anti-rat IgG (Amersham International, Little Chalfont, United Kingdom), diluted 1:50 in 0.1% BSA-C in PBS, for 4 h at 37°C. After short washes in PBS and distilled water, labeled grids were post-stained with uranyl acetate for 15 s.

The specificity of the immunogold labeling procedures was tested by several negative controls. Negative controls were treated either with pre-immune serum instead of the primary antibody and with non-specific secondary antibody (goat anti-mouse IgG). Negative controls for gibberellic acid revealed that no labeling occurred on the section when they were treated with pre-immune serum instead of the primary antibody (Supplementary Figure S2A) and with non-specific secondary antibody (Supplementary Figure S2B).

Nodule tissues were analyzed in a JEM–1400 EM transmission electron microscope (JEOL Ltd., Tokyo, Japan) at 80 kV. Electron micrographs were obtained by Veleta CCD camera (Olympus, Münster, Germany). Micrographs of randomly photographed immunogold-labeled sections were digitized, and gold particles were counted in visually identified cell structures. For statistical analysis, at least 10 different samples of root nodules and at least 50 sectioned symbiosomes were examined for SGE or mutant SGEFix^-^-3 (*sym26*). The SGEFix^-^-3 (*sym26*) mutant was chosen as an example of mutants with premature degradation of symbiotic structures (early senescence phenotype) for comparison with wild-type nodules. Previously, we demonstrated that early senescence is more pronounced in this mutant (Serova et al., 2018). Morphometrical data were obtained as described previously (Ivanova et al., 2015). Briefly, the number of gold particles per unit area was calculated. The areas and the number of gold particles were measured using software Zen 2 Core version 2.5 (Carl Zeiss). The data are presented as the number of gold particles/μm^2^. Data were analyzed by one-way ANOVA using the software SigmaPlot for Windows version 12.5 (Systat Software, Inc., San Jose, CA, United States). Means were separated by the Tukey multiple range test (*P*-value ≤ 0.001).

### Pharmacological Treatment

GA_3_ treatment was carried out for evaluation of GA action on pea nodule senescence. After inoculation with rhizobia, 100 ml of a 10^-6^ M GA_3_ (Sigma-Aldrich) aqueous solution was applied into substrate to SGE plants every 3 days until plants were harvested. Control plants were watered without GA_3_. Nodules were harvested for expression and light microscopy analyses. Photographs of pea nodules were made with a SteREO Lumar.V12 stereomicroscope equipped with an Axiocam ICc 1 video camera (Carl Zeiss).

#### Quantitative Measurements of Nodulation

GA_3_-treated and untreated pea plants were harvested for quantitative measurement. The shoots and roots were separated, and cotyledons were removed. Nodules on the primary and secondary roots were removed with a blade and counted. For weight measurements, separated shoots, roots, and nodules were dried at 42°C for 3 days. Experiments were performed with 9–16 plants per GA_3_-treated and untreated variants. Statistically significant differences were calculated using one-way ANOVA at *P*-value ≤ 0.01.

The sizes of nodules and senescence zones were measured with AxioVision Rel. 4.8 software (Carl Zeiss). Pictures of separate nodules of GA_3_-treated and untreated plants were taken as described above. Supplementary Figure S3 illustrates the selection and measurement of nodule projection areas using the example of analysis for a nodule of untreated plant at 6 WAI. Projection areas were measured as average projection area of nodules from the main root of a single plant. Experiments were performed with 8–12 plants per GA_3_-treated and untreated variants. Statistically significant differences were calculated using one-way ANOVA at *P*-value ≤ 0.01.

#### Light Microscopy Analysis of Nodules of GA_3_-Treated and Untreated Plants

Fixation of pea nodules for light microscopy was the same as described above. Samples were subsequently dehydrated with a series of ethanol solutions in water and embedded in Steedman’s wax as previously described (Serova et al., 2017). Sections of 10 μm were obtained with a HM360 microtome (Microm) and placed on slides in a few drops of water. After drying at 28°C for 30 min, slices were de-waxed in ethanol solutions and placed in PBS (Serova et al., 2017). Slices were then stained with toluidine blue (0.1% solution in PBS) for 10 min and washed in PBS (two times for 10 min). Sections were mounted in PBS.

Light microscopy analysis of nodule sections of GA_3_-treated and untreated plants was carried out with Axio Imager.Z1 (Carl Zeiss). Photographs were taken with a microscope camera Axiocam 506 color (Carl Zeiss) and analyzed using ZEN 2 core SP1 software (Carl Zeiss).

#### Expression Analysis of Senescence-Associated Genes

Primers for selected senescence-associated marker genes encoding cysteine protease 1 and 15a (*PsCyp1*, *PsCyp15a*), thiol protease (*PsTPP*), bZIP transcription factor (*PsATB2*), enzymes of ethylene (*PsACS2*, *PsACO1*) and ABA (*PsAO3*) biosynthesis, and an enzyme of bioactive GAs deactivation (*PsGA2ox1*) were designed previously (Serova et al., 2017). In addition, primers for a GA biosynthesis gene (*PsGA20ox1*) designed by Weston et al. (2008) were used.

RNA was extracted from 2, 4, and 6 WAI nodules of GA_3_-treated and untreated SGE as described above (see section “Expression Analysis of Genes Associated With GA Metabolism in SGE and Mutant Lines”). Reverse transcription and relative real-time PCR were performed in accordance with the previous description (see section “Expression Analysis of Genes Associated With GA Metabolism in SGE and Mutant Lines”).

## Results

### Expression Analysis of Genes Encoding Enzymes Involved in Biosynthesis and Deactivation of GAs

**FIGURE 1:**
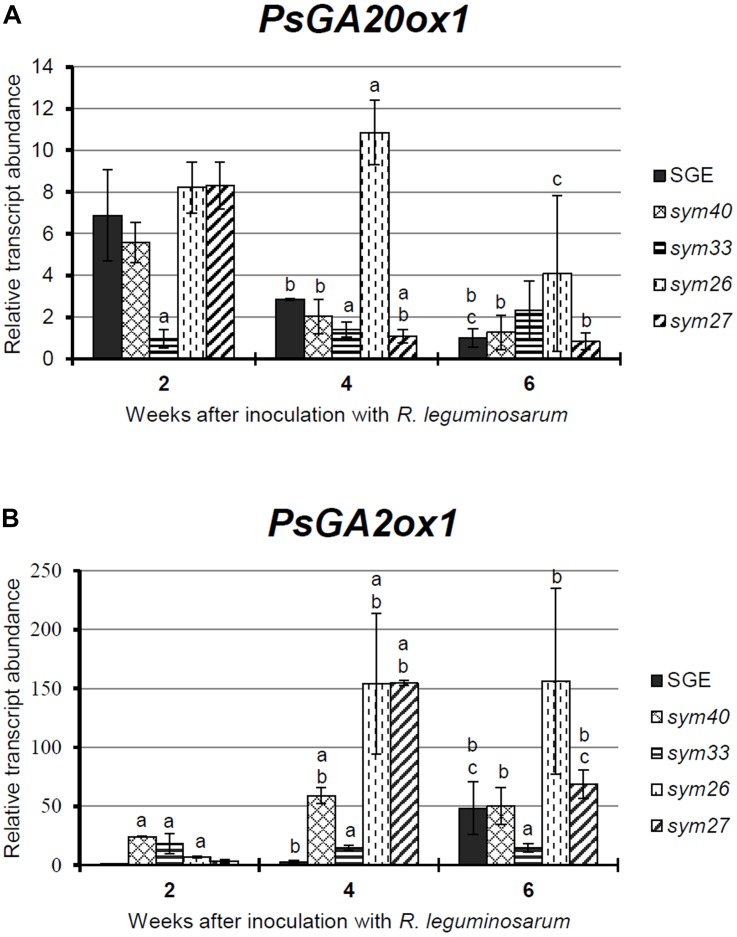
Relative transcript levels of *PsGA20ox1* **(A)** and *PsGA2ox1* **(B)** genes in nodules of wild-type SGE and corresponding pea mutants SGEFix^-^-1 (*sym40*), SGEFix^-^-2 (*sym33*), SGEFix^-^-3 (*sym26*), and SGEFix^-^-7 (*sym27*) at 2, 4, and 6 weeks after inoculation (WAI). *Letters* indicate significant differences (one-way ANOVA, *P*-value ≤ 0.05) a, from wild-type SGE at one time point; b, within genotype compared with 2 WAI; c, within genotype compared with 4 WAI.

To study the involvement of GAs in senescence of pea symbiotic nodules, an expression analysis of one GA biosynthesis gene (*PsGA20ox1*) and one GA deactivation gene (*PsGA2ox1*) was carried out in SGE and the mutant lines (Figure 1).

The transcript level of *PsGA20ox1* was down-regulated due to the aging of the wild-type and the mutant nodules with the exception of the mutant SGEFix^-^-2 (*sym33*), which showed statistically insignificant differences (Figure 1A). In the wild-type nodules, a significant (6.8-fold) decrease in transcript abundance was observed from 2 to 6 WAI. In 2- and 4-week-old nodules of the mutant SGEFix^-^-2 (*sym33*), the *PsGA20ox1* mRNA level was significantly reduced in comparison with the wild-type. In the mutant SGEFix^-^-3 (*sym26*), a slight down-regulation was detected from 4 to 6 WAI only. Furthermore, in 4-week-old nodules of the mutant SGEFix^-^-3 (*sym26*), the *PsGA20ox1* mRNA level was 3.8-fold higher than that in the wild-type. A significant (7.6-fold) down-regulation of the transcript level was observed in the mutant SGEFix^-^-7 (*sym27*) already at 4 WAI. In addition, *PsGA20ox1* transcript abundance was 2.6-fold lower in 4-week-old nodules of the mutant SGEFix^-^-7 (*sym27*) than in those of the wild-type. The difference in transcript levels in the nodules of the mutant SGEFix^-^-1 (*sym40*) from the wild-type was insignificant.

During aging of wild-type nodules, *PsGA2ox1* transcript abundance was significantly (48.4-fold) up-regulated at 6 WAI only. In contrast, the mutants SGEFix^-^-3 (*sym26*) and SGEFix^-^-7 (*sym27*) showed 23.5- and 49.4-fold elevation of the expression level, respectively, already at 4 WAI. However, in the case of the mutant SGEFix^-^-7 (*sym27*), a decrease in *PsGA2ox1* transcript abundance was detected at 6 WAI. In 4-week-old nodules of the mutant SGEFix^-^-1 (*sym40*), an increase in *PsGA2ox1* mRNA was less pronounced. *PsGA2ox1* transcript abundance was significantly higher in the mutants SGEFix^-^-1 (*sym40*), SGEFix^-^-3 (*sym26*), and SGEFix^-^-7 (*sym27*) than in the wild-type nodules at 4 WAI. The expression level of *PsGA2ox1* gene was slightly higher than in the wild-type in 4-week-old nodules of the mutant SGEFix^-^-2 (*sym33*). However, changes in the transcript level of the mutant SGEFix^-^-2 (*sym33*) were statistically insignificant.

Overall, the expression of the GA biosynthesis gene *PsGA20ox1* was decreased, while the expression of the GA deactivation gene *PsGA2ox1* was increased during aging of the pea nodules of wild-type and early senescent mutants.

### GA_3_ Immunolocalization in Wild-Type and Mutant Nodules

**FIGURE 2:**
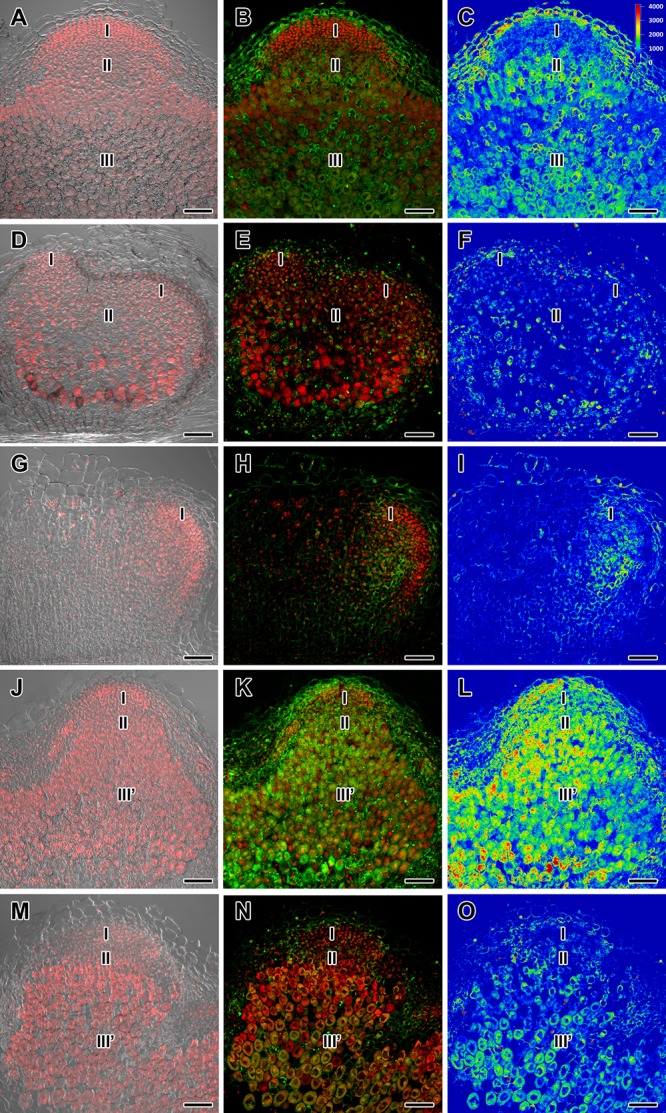
Immunolocalization of gibberellin (GA_3_) in nodules of wild-type SGE **(A–C)** and corresponding pea mutants SGEFix^-^-1 (*sym40*) **(D–F)**, SGEFix^-^-2 (*sym33*) **(G–I)**, SGEFix^-^-3 (*sym26*) **(J–L)**, and SGEFix^-^-7 (*sym27*) **(M–O)** at 2 weeks after inoculation. Zones of nodules are designated by Roman numerals: I – meristem, II – infection zone, III – fixation zone, III’ – zone corresponding to nitrogen fixation zone in wild-type. A differential interference contrast microscopy image merged with laser scanning confocal microscopy image in red channel **(A,D,G,J,M)**. Merged images of laser scanning confocal microscopy in green and red channels **(B,E,H,K,N)**. Heat map provides a color code of fluorescence signal intensities **(C,F,I,L,O)**. Visualization of GA_3_ by the Alexa Fluor 488 conjugated secondary antibody (green), nuclei, and bacteria stained with propidium iodide (red). Scale bar = 100 μm.

**FIGURE 3:**
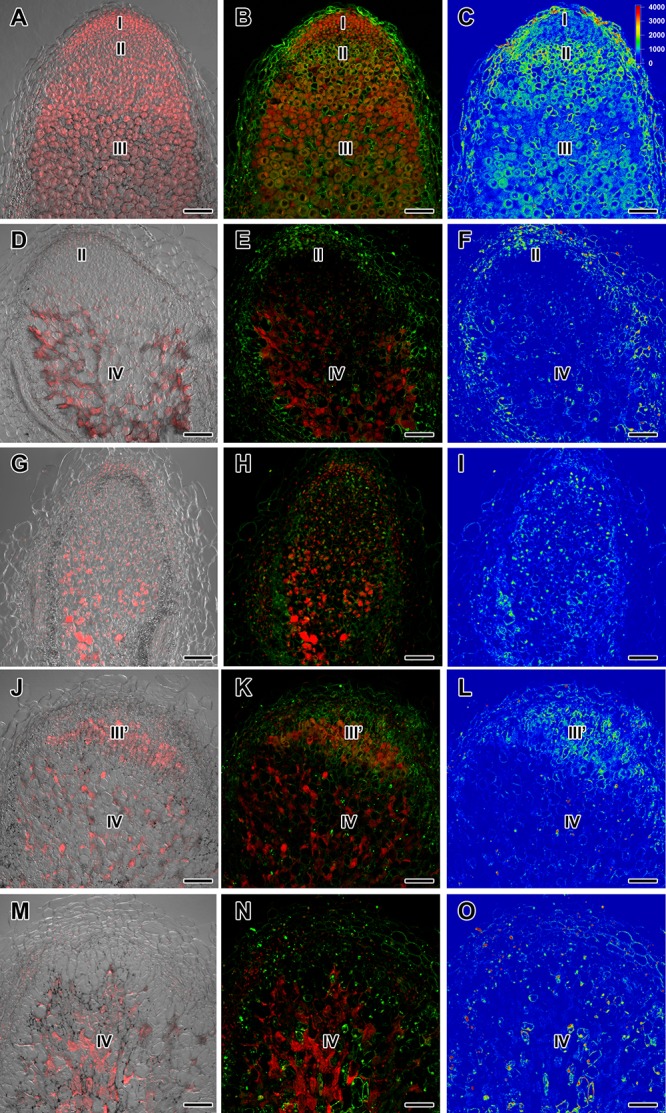
Immunolocalization of gibberellin (GA_3_) in nodules of wild-type SGE **(A–C)** and corresponding pea mutants SGEFix^-^-1 (*sym40*) **(D–F)**, SGEFix^-^-2 (*sym33*) **(G–I)**, SGEFix^-^-3 (*sym26*) **(J–L),** and SGEFix^-^-7 (*sym27*) **(M–O)** at 4 weeks after inoculation. Zones of nodule are designated by Roman numerals: I – meristem, II – infection zone, III – fixation zone, III’ – zone corresponding to nitrogen fixation zone in wild-type, IV – senescence zone. A differential interference contrast microscopy image merged with laser scanning confocal microscopy image in red channel **(A,D,G,J,M)**. Merged images of laser scanning confocal microscopy in green and red channels **(B,E,H,K,N)**. Heat map provides a color code of fluorescence signal intensities **(C,F,I,L,O)**. Visualization of GA_3_ by the Alexa Fluor 488 conjugated secondary antibody (green), nuclei, and bacteria stained with propidium iodide (red). Scale bar = 100 μm.

**FIGURE 4:**
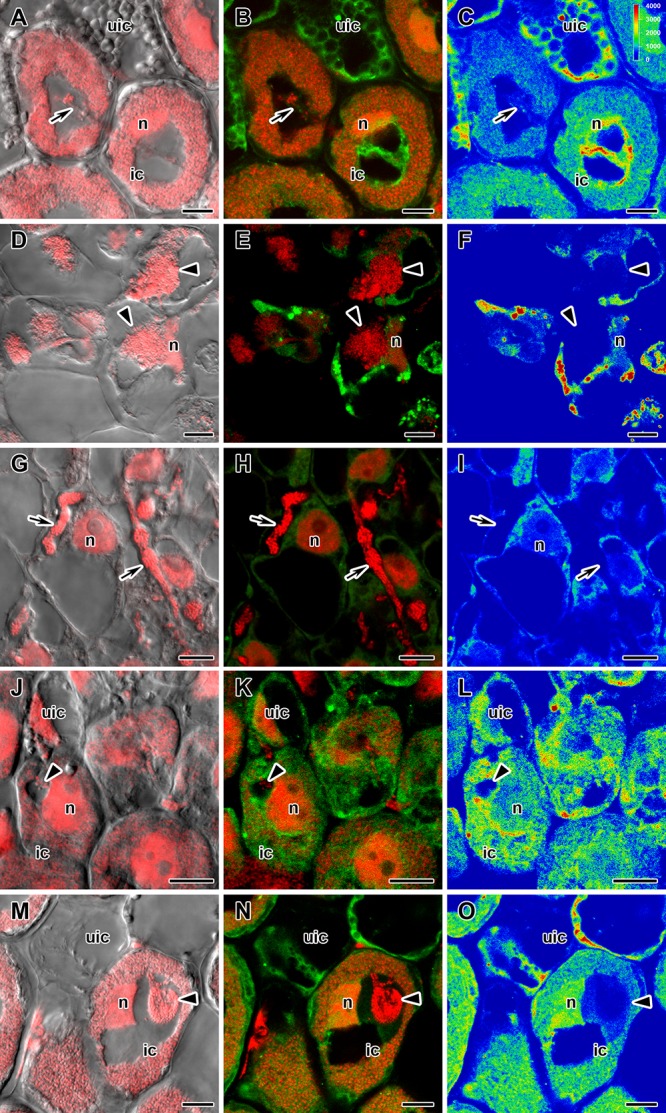
Immunolocalization of gibberellin (GA_3_) in cells in central part of nodules of wild-type SGE **(A–C)** and corresponding pea mutants SGEFix^-^-1 (*sym40*) **(D–F)**, SGEFix^-^-2 (*sym33*) **(G–I)**, SGEFix^-^-3 (*sym26*) **(J–L)**, and SGEFix^-^-7 (*sym27*) **(M–O)** at 2 weeks after inoculation. ic, infected cell; uic, uninfected cell; n, nucleus. Arrow indicates infection thread, arrowhead indicates infection droplet. A differential interference contrast microscopy image merged with laser scanning confocal microscopy image in red channel **(A,D,G,J,M)**. Merged images of laser scanning confocal microscopy in green and red channels **(B,E,H,K,N)**. Heat map provides color code of fluorescence signal intensities **(C,F,I,L,O)**. Visualization of GA_3_ by the Alexa Fluor 488 conjugated secondary antibody (green), nuclei, and bacteria stained with propidium iodide (red). Scale bar = 10 μm.

To complement the data obtained by expression analysis of GA metabolism genes, GA_3_ immunolocalization was carried out in wild-type and mutant nodules of different ages (Figure 2–4 and Supplementary Figures S4–S6).

In 2- and 4-week-old wild-type nodules, GA_3_ labeling was detected in cells from the meristem, and in the infection and nitrogen fixation zones (Figure 2A–C, 3A–C and Supplementary Figure S4). The GA_3_ label was observed in both infected and uninfected cells; in the latter, it was associated with cytoplasm surrounding starch granules (Figure 4A–C). In wild-type nodules, a high signal of GA_3_ labeling was also detected in nuclei, mostly in cells from the nitrogen fixation zone (Figure 4A–C and Supplementary Figures S1G–L). The intensity of labeling was much lower in 6-week-old wild-type nodules (Supplementary Figures S5A–C,G–I), especially in the senescence zone (Supplementary Figures S5D–F). Traces of GA_3_ label were detected in senescent uninfected cells (Supplementary Figures S5J–L). In 2-week-old nodules of the mutant SGEFix^-^-1 (*sym40*), a high intensity of labeling was observed in nuclei and cytoplasm, but was absent in infection threads and infection droplets (Figure 4D–F). Whole nodule intensity of labeling was lower in the mutant SGEFix^-^-1 (*sym40*) than in the wild-type (Figure 2D–F). In 4-week-old nodules of the mutant SGEFix^-^-1 (*sym40*), the intensity of GA_3_ labeling was significantly decreased (Figure 3D–F); traces of the GA_3_ label were observed in nuclei and cytoplasm, particularly at the cell periphery (Supplementary Figures S6A–C). In nodules of the mutant SGEFix^-^-2 (*sym33*), the lowest level of GA_3_ was observed (Figure 2G–I). The GA_3_ label was detected mainly in nuclei and cytoplasm, but was absent in infection threads (Figure 4G–I). In 4-week-old mutant nodules, a high level of fluorescence was associated with nuclei (Figure 3G–I and Supplementary Figures S6D–F). A high intensity of fluorescence was detected in the mutants SGEFix^-^-3 (*sym26*) (Figure 2J–L) and SGEFix^-^-7 (*sym27*) (Figure 2M–O) in 2-week-old nodules. In SGEFix^-^-3 (*sym26*), it was even higher than that of the wild-type nodules of the same age. In the mutants SGEFix^-^-3 (*sym26*) and SGEFix^-^-7 (*sym27*), the maximum GA_3_ signal was observed in infected and uninfected cells of regions corresponding to the nitrogen fixation zone of wild-type nodules (Figure 4J–O). The amount of GA_3_ was significantly decreased in the senescence zone occupying the dominant part of 4-week-old mutant nodules (Figure 3J–O). Trace levels of GA_3_ label were observed in uninfected cells (Supplementary Figures S6G–L).

Thus, a decline in the amount of detectable GA_3_ during aging of pea symbiotic nodules of wild-type and mutant lines and its distribution in different histological nodule zones were demonstrated.

### Immunogold Localization of GA_3_ in Nodules of Wild-Type SGE and Mutant SGEFix^-^-3 (*sym26*)

**FIGURE 5:**
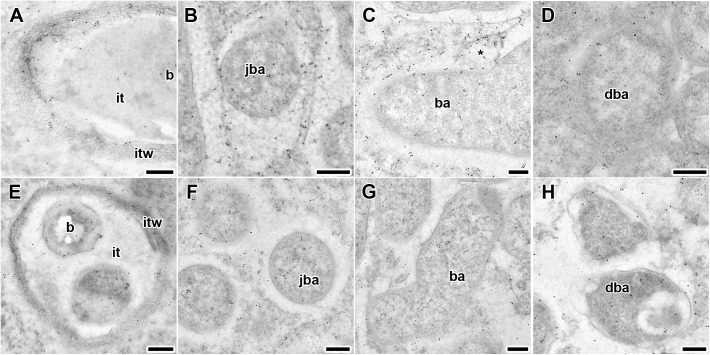
Immunogold localization of gibberellin (GA_3_) in nodules of wild-type SGE **(A–D)** and mutant SGEFix^-^-3 (*sym26*) **(E–H)** at 2 weeks after inoculation. Secondary goat anti-rat IgG MAb conjugated to 10 nm diameter colloidal gold was used. it, infection thread; itw, infection thread wall; b, bacterium; ba, bacteroid; jba, juvenile bacteroid; dba, degrading bacteroid; asterisk indicates vesicle with GA_3_ label. **(A,E)** Infection threads, **(B,F)** juvenile symbiosomes, **(C,G)** mature symbiosomes, **(D,H)** senescent symbiosomes. Scale bar **(A,E)** = 500 nm, **(B–D,F–H)** = 200 nm.

To compare the localization of GA_3_ in the infection structures in 2-week-old nodules, immunogold localization of GA_3_ was carried out in the wild-type and SGEFix^-^-3 (*sym26*) nodules (Figure 5).

Gold particles were observed in nuclei, vacuoles, and plastids, and were very abundant in cytoplasm (data not shown). Rare gold particles were observed in the matrix of infection threads in wild-type (Figure 5A) and SGEFix^-^-3 (*sym26*) (Figure 5E) as well as in the infection droplets (Table 2). Gold particles were mainly found in the infection thread walls and in bacteria embedded in the infection thread matrix (Figure 5A,E). With respect to symbiosomes, numerous gold particles were observed mainly in bacteroids of both SGE and mutant SGEFix^-^-3 (*sym26*) (Figure 5B–D,F–H), although some labeling was also found in the symbiosome membrane especially in the juvenile symbiosomes in nodules of wild-type SGE (Figure 5B). In nodules of SGE, some vesicles with GA_3_ label were found in the peribacteroid spaces (Figure 5C). The highest amount of gold particles was observed in juvenile symbiosomes in both analyzed genotypes (Figure 5B,F and Table 2). In mature symbiosomes and especially in senescent symbiosomes in nodules of SGE and mutant SGEFix^-^-3 (*sym26*), the amount of gold particles of the GA_3_ label was lower than that in juvenile symbiosomes (Figure 5C,D,G,H and Table 2). It is necessary to note that the amount of gold particles of the GA_3_ label was lower in symbiosomes of the mutant SGEFix^-^-3 (*sym26*) than in wild-type nodules (Table 2).

**Table 2:** Distribution of gold particles in 2-week-old nodules of pea wild-type SGE and mutant SGEFix^-^-3 (*sym26*) (immunogold localization).

Genotype	Localization	Mean value	*SE*
SGE	Juvenile symbiosomes	61.25^ad^	2.73
	Mature symbiosomes	43.03^bd^	2.46
	Senescent symbiosomes	19.90^cd^	2.94
	Infection threads and infection droplets	11.15^f^	2.78
SGEFix^-^-3 (*sym26*)	Juvenile symbiosomes	40.95^ae^	6.29
	Mature symbiosomes	32.28^be^	3.38
	Senescent symbiosomes	24.85^ce^	2.98
	Infection threads and infection droplets	15.36^f^	2.23


*Results are presented as the number of gold particles/μm^2^. Mean value ± SE (*n* = 30–60) are shown. *Letters* indicate statistically significant differences by the Tukey multiple range test (*P*-value ≤ 0.001): a, between juvenile symbiosomes of wild type and the mutant; b, between mature symbiosomes of wild type and the mutant; c, between senescent symbiosomes of wild type and the mutant; d, between juvenile, mature, and senescent symbiosomes in wild type; e, between juvenile, mature, and senescent symbiosomes in the mutant; f, between infection threads and infection droplets in wild type and the mutant*.

Thus, it was shown that with an increase in the age of wild-type and mutant nodules, the amount of GA_3_ decreased in symbiosomes.

### Analysis of Nodules of SGE Treated With Exogenous GA_3_ Relative to Untreated Plants

**FIGURE 6:**
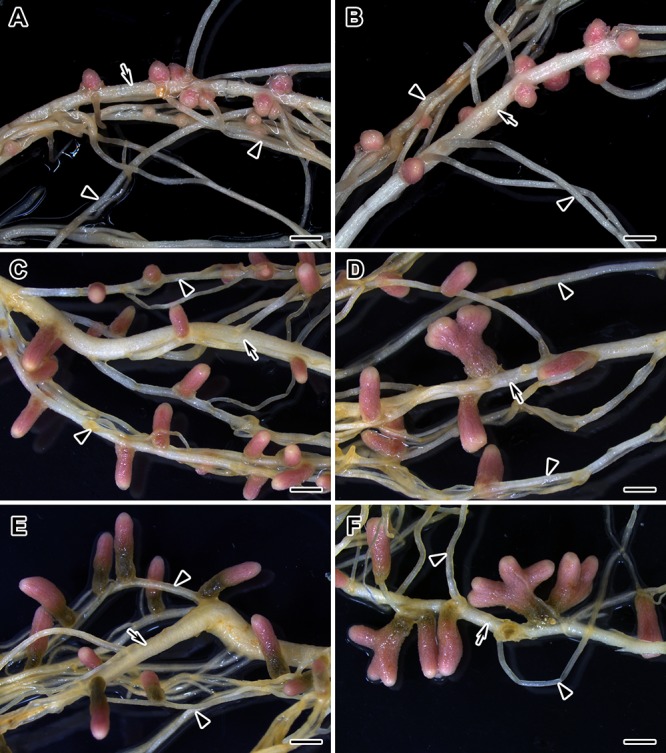
Nodulated main and lateral roots of wild-type SGE pea plants untreated **(A**,**C**,**E)** and treated with exogenous gibberellin (GA_3_) (**B**,**D**,**F)** at 2, 4, and 6 weeks after inoculation. Arrow indicates main roots; arrowheads indicate lateral roots. Scale bar = 2 mm.

**FIGURE 7:**
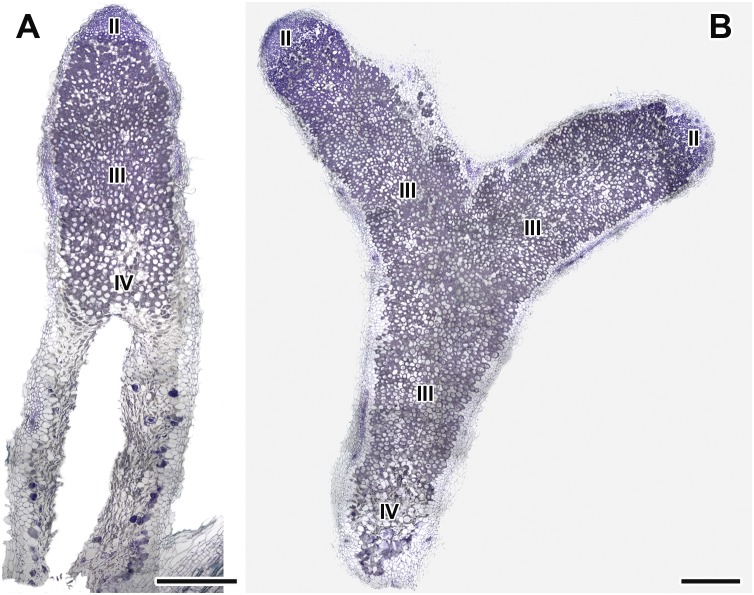
Histological organization of nodules in wild-type SGE pea plants untreated **(A)** and treated with exogenous gibberellin (GA_3_) **(B)** at 6 weeks after inoculation. Zones in nodules are designated by Roman numerals: II – infection zone, III – fixation zone, IV – senescence zone. Sections were stained with toluidine blue. Scale bar = 500 μm.

**FIGURE 8:**
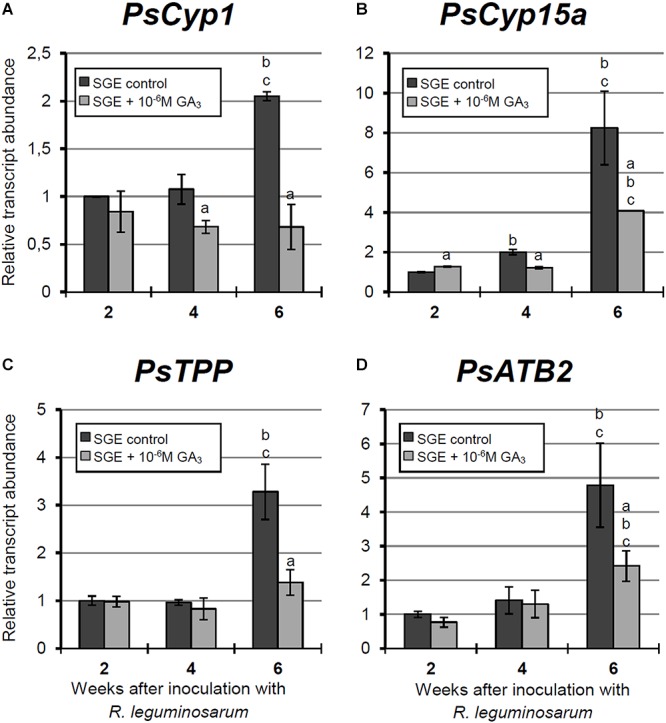
Relative expression of **(A–D)** *PsCyp1*, *PsCyp15a*, *PsTPP*, and *PsATB2* genes in nodules of GA_3_-treated and untreated wild-type SGE at 2, 4, and 6 weeks after inoculation (WAI). *Letters* indicate significant differences (one-way ANOVA, *P*-value ≤ 0.05, *n* = 3) a, from untreated plants at one time point; b, within variant (GA_3_-treated or untreated) compared with 2 WAI; c, within variant (GA_3_-treated or untreated) compared with 4 WAI.

**FIGURE 9:**
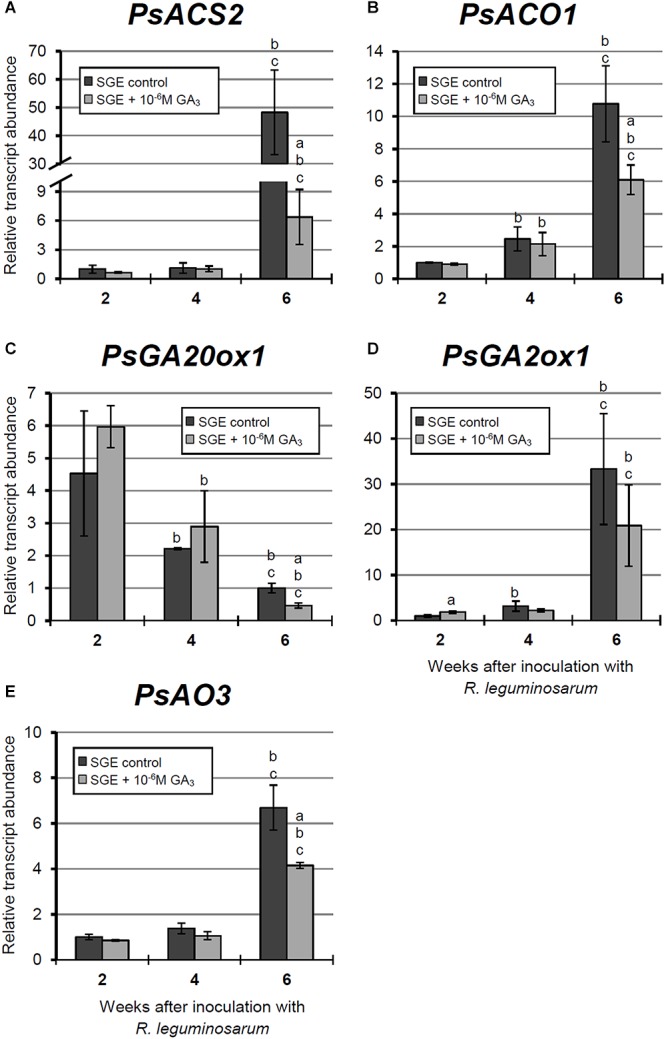
Relative expression of **(A–E)** *PsACS2*, *PsACO1*, *PsGA20ox1*, *PsGA2ox1*, and *PsAO3* genes in nodules of GA_3_-treated and untreated wild-type SGE at 2, 4, and 6 weeks after inoculation (WAI). *Letters* indicate significant differences (one-way ANOVA, *P*-value ≤ 0.05, *n* = 3) a, from untreated plants at one time point; b, within variant (GA_3_-treated or untreated) compared with 2 WAI; c, within variant (GA_3_-treated or untreated) compared with 4 WAI. Breaks of histogram bars indicate a change in scale.

The involvement of bioactive GAs in senescence of pea symbiotic nodules was also measured via GA_3_ treatment of wild-type plants (Figure 6). A quantitative analysis of 2-, 4-, and 6-week-old nodules of GA_3_-treated and untreated plants was carried out (Tables 3, 4). Additionally, an analysis of the effect of GA_3_-treatment on the histological structure of the symbiotic nodule was performed (Figure 7). To identify the involvement of bioactive GAs in senescence of pea nodules at the transcriptional level, the analysis of senescence-associated genes was carried out in nodules of GA_3_-treated and untreated plants at 2, 4, and 6 WAI (Figure 8, 9).

**Table 3:** Shoot, root, and nodule dry weight (DW), nodule number per plant, percentage of nodules with meristem bifurcation and nodules without senescence zone of GA_3_-treated and untreated wild-type SGE at 2, 4, and 6 weeks after inoculation (WAI).

Treatment	WAI	Shoot DW, mg	Root DW, mg	Total nodule DW, mg	Average nodule DW, mg	Number of nodules per plant	Percentage of nodules with meristem bifurcation	Percentage of nodules without senescence zone
SGE	2	61.6 ± 5.97	30.64 ± 2.47	2.11 ± 0.26	0.1 ± 0.008	21 ± 1.81	ND	ND
	4	148.65 ± 11.39^b^	48.82 ± 4.55^b^	15.03 ± 1.19^b^	0.27 ± 0.012^b^	54.75 ± 3.52b	9.13 ± 1.91	ND
	6	659.62 ± 45.17^bc^	118.52 ± 14.18^bc^	21.02 ± 2.48^b^	0.28 ± 0.016^b^	78.45 ± 6.68bc	3.63 ± 0.74	13.92 ± 2.34
SGE + 10^-6^M GA_3_	2	77.24 ± 9.79	36.17 ± 3.28	3.24 ± 0.52	0.21 ± 0.018^a^	13.45 ± 1.63a	ND	ND
	4	244 ± 15.58^ab^	52.38 ± 4.35^b^	14.32 ± 1.34^b^	0.67 ± 0.085^ab^	22.64 ± 1.45ab	32.52 ± 4.86^a^	ND
	6	747.44 ± 96.54^bc^	109.03 ± 14.35^bc^	17.7 ± 3.03^b^	0.64 ± 0.15^ab^	36.78 ± 5.94ab	23.01 ± 4.57^a^	40.26 ± 3.96^a^


*Plants were inoculated with *R. leguminosarum* 3841. Results are means ± SE (*n* = 9–16). *Letters* indicate statistically significant differences (*P*-value ≤ 0.01): a, from untreated plants at one time point; b, within variant (GA_3_-treated or untreated) compared with 2 WAI; c, within variant (GA_3_-treated or untreated) compared with 4 WAI. ND, no data*.

**Table 4:** Projection areas of whole nodule and senescence zone of GA_3_-treated and untreated wild-type SGE pea plants at 2, 4, and 6 weeks after inoculation (WAI).

Treatment	WAI	Projection area of whole nodule, mm^2^	Projection area of senescence zone, % of whole nodule projection area
SGE	2	0.91 ± 0.05	ND
	4	2.82 ± 0.12^b^	ND
	6	3.56 ± 0.17^bc^	62.4 ± 2.47
SGE + 10^-6^ M GA_3_	2	1.63 ± 0.07^a^	ND
	4	5.48 ± 0.35^ab^	ND
	6	5.84 ± 0.76^ab^	34.0 ± 3.08^a^


*Plants were inoculated with *R. leguminosarum* 3841. Results are means ± SEM (*n* = 8–13). *Letters* indicate statistically significant differences (*P-*value ≤ 0.01): a, from untreated plants at one time point; b, within variant (GA_*3*_-treated or untreated) compared with 2 WAI; c, within variant (GA_*3*_-treated or untreated) compared with 4 WAI. ND, no data*.

#### Quantitative Measurements of Nodulation of GA_3_-Treated and Untreated Plants

During nodule aging, an increase in dry weight (DW) and size of nodules was observed in both GA_3_-treated and untreated plants (Tables 3, 4). In the untreated plants, the average nodule DW and the projection area of the whole nodule were increased 2.8 and 4 times, respectively, during aging from 2 to 6 WAI (Figure 6A,C,E and Tables 3, 4). In 6-week-old untreated plants, 10- and 3.7-fold increases in the total nodule DW and the number of nodules, respectively, were observed relative to 2-week-old plants (Table 3). A low degree (9.13 and 3.63%) of meristem bifurcation was detected in 4- and 6-week-old nodules of untreated plants, respectively (Figure 6C,E and Table 3). At 6 WAI, the senescence zone dominated in the nodules of untreated plants and occupied approximately 62% of the entire nodule (Figure 6E and Table 4).

In the case of GA_3_-treatment, 3- and 3.6-fold increases in the average nodule DW and projection area of the whole nodule, respectively, were observed at 6 WAI, in comparison with 2 WAI (Figure 6B,D,F and Tables 3, 4). In GA_3_-treated plants, the total nodule DW and the number of nodules were 5.4- and 2.7-times higher, respectively, during aging from 2 to 6 WAI (Table 3). Pronounced meristem bifurcation (32.52 and 23.01%) was observed in 4- and 6-week-old nodules of GA_3_-treated plants, respectively (Figure 6D,F and Table 3). Despite the presence of the senescence zone occupying approximately 34% of the entire nodule, the biggest part of the nodules in GA_3_-treated plants was represented by the nitrogen fixation zone (Figure 6F and Table 4).

The average DW and projection area of whole 2-week-old nodules of GA_3_-treated plants were 2.1 and 1.8 times, respectively, larger than those of nodules of untreated plants (Tables 3, 4). At 2 WAI, visual manifestation of the senescence zone (green color) was absent in nodules of GA_3_-treated and untreated plants (Figure 6A,B). At 4 WAI, the average nodule DW and projection area of nodules of plants treated with exogenous GA_3_ were 2.5 and 2 times larger, respectively, than the nodules of the untreated plants (Tables 3, 4). In addition to the increase in size, the percentage of nodules exhibiting meristem bifurcation was 3.6 times greater in GA_3_-treated plants than in untreated plants at 4 WAI (Figure 6C,D and Table 3). At 6 WAI, GA_3_-treated plants also had a high percentage of nodules with branching due to meristem bifurcation being 6.3 times higher than that in untreated plants (Figure 6F and Table 3). At 6 WAI, nodules of plants treated with exogenous GA_3_ were 2.3 and 1.6 times (average nodule DW and nodule projection area, respectively) larger than the nodules of the untreated plants (Figure 6F and Tables 3, 4). In addition, in 6-week-old GA_3_-treated plants, the number of nodules without visible signs of senescence was 2.8 times higher than that of untreated plants (Table 3). Also, it was noted that the number of nodules of plants treated with exogenous GA_3_ was 1.5, 2.4, and 2.1 times smaller than that of the nodules of untreated plants at 2, 4, and 6 WAI, respectively (Table 3). In GA_3_-treated plants, a 1.6-fold increase in shoot DW was observed at 4 WAI relative to the control (Table 3). At 2 and 6 WAI, these differences were insignificant.

#### Microscopic Analysis of Nodule Structure of GA_3_-Treated and Untreated Plants

Longitudinal toluidine blue-stained sections of nodules of untreated and GA_3_-treated plants were compared at 6 WAI. The nodules of untreated and GA_3_-treated plants had the typical zonation of indeterminate nodules (Figure 7). In 6-week-old nodules of untreated plants, the senescence zone, which was characterized by flattened cells, loss of cell rigidity and cellular integrity, and plant cell wall breakage, occupied most of the nodule and contained a cavity due to complete decay of nodule cells (Figure 7A). While nodules of GA_3_-treated plants had a vast nitrogen fixation zone, the senescence zone contained both infected and uninfected cells and occupied a smaller part than that in the nodules of untreated plants (Figure 7B).

#### Expression Analysis of Senescence-Associated Genes in Wild-Type Nodules Treated With Exogenous GA_3_

In this study, an analysis of the expression of senescence-associated genes (Serova et al., 2017, 2018) and genes encoding enzymes of GA biosynthesis (*PsGA20ox1*) and deactivation (*PsGA2ox1*) was carried out. Changes in the mRNA levels of these genes during GA_3_ treatment allowed us to evaluate the possible effect of GAs on nodule senescence at the level of transcription.

A significant increase in the expression levels of selected cysteine protease genes was observed during nodule aging of the untreated plants. However, elevation of transcript abundance in nodules of GA_3_-treated plants was less pronounced (Figure 8A–C). In 6-week-old nodules of the untreated plants, expression levels of *PsCyp1*, *PsCyp15a*, and *PsTPP* genes were significantly increased relative to 2-week-old nodules, while in nodules of GA_3_-treated plants, no statistically significant differences in *PsCyp1* and *PsTPP* transcript abundances were detected during nodule aging (Figure 8A,C). At 6 WAI, an up-regulation in *PsCyp15a* mRNA was detected in nodules of GA_3_-treated plants; however, the expression level was two times lower than in the untreated control (Figure 8B). In the case of *PsCyp1* and *PsTPP* genes, expression levels in mature nodules of GA_3_-treated plants were 3- and 2.4-times lower, respectively, than in the untreated plants (Figure 8A,C).

A significant up-regulation of *PsATB2* transcript abundance was observed in the nodules of untreated plants from 4 to 6 WAI. A less pronounced elevation of expression was detected during aging of nodules of GA_3_-treated plants, in comparison with the untreated control. Also, it was noted that at 6 WAI, *PsATB2* transcript abundance was two times lower in nodules of GA_3_-treated plants than in the untreated plants (Figure 8D).

During nodule aging of the untreated plants, the expression levels of genes encoding key enzymes of ethylene biosynthesis, *PsACS2* and *PsACO1*, were significantly increased from 2 to 6 WAI. A less pronounced up-regulation of *PsACS2* and *PsACO1* mRNA levels was observed in 6-week-old nodules of GA_3_-treated plants in comparison with 2-week-old nodules. At 6 WAI, *PsACS2* and *PsACO1* expression levels were 7.6- and 1.7-times lower, respectively, in nodules of GA_3_-treated plants than in those of untreated plants (Figure 9A,B).

A significant decrease in the expression level of the *PsGA20ox1* gene was observed during nodule aging in both untreated and GA_3_-treated plants. It is worth noting that in 6-week-old nodules of GA_3_-treated plants, *PsGA20ox1* transcript abundance was 2.15-times lower than that in the nodules of untreated plants (Figure 9C). At 6 WAI, a significant elevation in the expression of *PsGA2ox1* was observed in the nodules of untreated and GA_3_-treated plants, relative to 2 WAI. However, the difference between nodules of untreated and GA_3_-treated plants at 6 WAI was not statistically significant. In 2-week-old nodules of GA_3_-treated plants, however, *PsGA2ox1* transcript abundance was slightly higher than in the nodules of untreated plants (Figure 9D).

Finally, up-regulated expression of *PsAO3*, the gene encoding an enzyme of the final stage of ABA biosynthesis, was detected in the nodules of untreated and GA_3_-treated plants from 2 to 6 WAI. In 6-week-old nodules of GA_3_-treated plants, *PsAO3* transcript abundance was slightly reduced relative to that in untreated plants (Figure 9E).

Thus, a down-regulation of senescence-associated genes, a decrease of senescence zone, an extension of nitrogen-fixation zone, an increase of nodule size, and the pronounced meristem bifurcation were observed in wild-type nodules treated with exogenous GA_3_ in comparison with the untreated plants.

## Discussion

In this study, we investigated the involvement of GAs in senescence of the symbiotic nodule using SGE and corresponding pea mutants blocked at different stages of nodule development. The mutant SGEFix^-^-1 (*sym40*) forms numerous white nodules with hypertrophied infection threads and droplets, and abnormal bacteroids (Tsyganov et al., 1998). White nodules colonized with “locked” suberinized infection threads are typical for SGEFix^-^-2 (*sym33*) (Tsyganov et al., 1998; Ivanova et al., 2015). SGEFix^-^-2 carries a weak allele of the gene *sym33* and manifests a leaky phenotype. Usually, bacteria are not released from these infection threads; however, in some cells or some nodules, bacterial release occurs (Voroshilova et al., 2001). The mutants SGEFix^-^-3 (*sym26*) and SGEFix^-^-7 (*sym27*) are characterized by premature degradation of symbiotic structures, with degradation more pronounced in the former than in the latter (Serova et al., 2018). Recently, we demonstrated activation of nodule senescence in nodules of all analyzed mutants (Serova et al., 2018).

**FIGURE 10:**
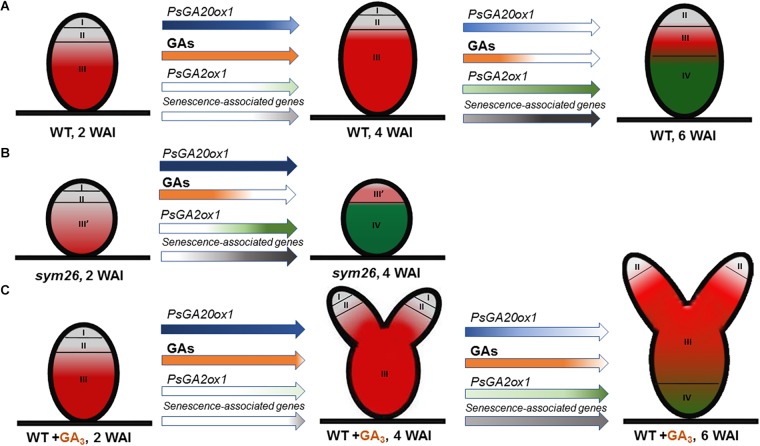
Scheme of endogenous gibberellin influence on nodule senescence in wild-type **(A)** and mutant SGEFix^-^-3 (*sym26*) nodules **(B)** and influence of exogenous GA_3_ on nodule senescence and meristem bifurcation in wild-type **(C)**. Zones of nodule are designated by Roman numerals: I – meristem (in gray), II – infection zone (in gray), III – fixation zone, (in red) III’ – zone corresponding to nitrogen fixation zone in wild-type (in red), IV – senescence zone (in green), WAI – weeks after inoculation. An increase in the color intensity of the arrows indicates elevated levels of gene expression, a decrease in color intensity indicates a reduction in expression levels. A decrease in orange intensity indicates a reduction in GA levels. The scheme is based on the results of this study and Serova et al. (2018).

Previously, an increase in the expression level of GA biosynthesis genes (*GA20ox*, *GA3ox*) during early stages of the development of both determinate (Kouchi et al., 2004; Lievens et al., 2005; Maekawa et al., 2009; Hayashi et al., 2012) and indeterminate (Larrainzar et al., 2015) nodules was shown. A decrease in the transcript abundance of the GA biosynthesis genes was observed in mature nodules (Lievens et al., 2005; Hayashi et al., 2012). In our study, a significant decline in the transcript level of the *PsGA20ox1* gene was detected in wild-type nodules at 4 and 6 WAI (Figure 10A). One of the mechanisms to maintain optimal concentrations of bioactive GAs is 2β-hydroxylation, which leads to a decrease in content of bioactive GAs (Thomas et al., 1999; Hedden and Thomas, 2012). *GA2ox* transcript levels were found to be elevated during the aging of symbiotic nodules of *M. truncatula* (Van de Velde et al., 2006) and pea (Serova et al., 2017) plants. Furthermore, *GA2ox* transcripts were detected in maturing nodules of *L. japonicus* (Kouchi et al., 2004). In this study, the *PsGA2ox1* mRNA level was elevated at 6 WAI (Figure 10A). Thus, we suggest that during nodule aging, bioactive GA levels decrease; maintaining the optimal concentration of bioactive GAs appears to occur via the down-regulation of its biosynthetic genes and the up-regulation of its deactivation genes. The results of expression analysis were complemented by those obtained from immunolocalization of bioactive GA_3_. In the wild-type plants, a high intensity of GA_3_ label was detected in 2-week-old nodules and was maintained in mature 4-week-old nodules, but it was significantly decreased in old 6-week-old nodules (Figure 10A). The high GA_3_ content in meristematic cells may indicate the involvement of GAs in cell cycle activation, cell division, and persistence of the nodule meristem. Previously, it was shown that GAs modulate cell cycle activity in Arabidopsis roots (Achard et al., 2009). In addition, pea mutants impaired in GA biosynthetic genes displayed decreased nodulation and aberrant nodule meristem formation (Ferguson et al., 2005, 2011).

A high intensity of GA_3_ labeling was detected in the infected cells in the nitrogen fixation zone, where it was associated mainly with cytoplasm and symbiosomes. In juvenile symbiosomes, the amount of label was higher than in mature and senescent symbiosomes. It is worth noting that McAdam et al. (2018) recently revealed that GAs promote the functioning of nitrogen-fixing nodules. However, the exact function of GAs in infected cell and symbiosome development merits elucidation. Previously, a negative impact of GAs on infection thread development was revealed (McAdam et al., 2018). In our study, we observed that the amount of GA_3_ in the infection threads was lower than in symbiosomes. However, GA_3_ was detected in infection thread walls. The function of GAs in infection thread development is currently unknown. It is interesting to note that GA involvement in facilitating bacterial release from the infection threads was recently suggested (Tatsukami and Ueda, 2016).

In uninfected cells, labeling of GA_3_ was associated with cytoplasm around starch granules. Ferguson and Mathesius (2003) suggested involvement of GAs in the hydrolysis of nodule starch through enhancement of α-amylase production. Substrates formed during this reaction might support the energetic requirements of rhizobia. In general, GAs may benefit both symbiotic partners through involvement in cell division and elongation, and by providing energy to support plant growth. GAs may enhance photosynthetic capacity, leading to an increase in photoassimilate content for growth and functioning of the symbiotic nodule (Ferguson and Mathesius, 2003). Six-week-old wild-type nodules contained a low amount of bioactive GA_3_ (Figure 10A). Traces of GA_3_ label were detected in the uninfected cells of the senescence zone; these cells might be degraded later that the infected cells of that same zone (Pladys et al., 1991).

Expression analysis of GA metabolism genes revealed down-regulation of a GA biosynthesis gene, *PsGA20ox1*, and significant up-regulation of a GA deactivation gene, *PsGA2ox1*, during nodule aging of the early senescent mutants SGEFix^-^-7 (*sym27*) and SGEFix^-^-3 (*sym26*), which confirms involvement of GAs in senescence of symbiotic nodules (Figure 10B). However, for mutant SGEFix^-^-3 (*sym26*), an increase in *PsGA20ox1* expression in 4-week-old nodules in comparison with 2-week-old nodules was observed. This increase is difficult to explain, but future elucidation of the gene *sym26* molecular product may be informative. Data obtained with GA_3_ immunolocalization coincided with the observed transcriptional patterns. In 2-week-old nodules of the mutants SGEFix^-^-3 (*sym26*) and SGEFix^-^-7 (*sym27*), the maximum GA_3_ signal was observed in cells of the region corresponding to the nitrogen fixation zone of the wild-type nodules (Figure 10B). In the vast senescence zone of 4-week-old nodules, the intensity of GA_3_ label was low and predominantly found in uninfected cells. Both mutants form nodules with morphologically differentiated bacteroids, which characterized with early senescence (Serova et al., 2018). Recently, it was shown that in defective nodules of GA-deficient mutant *na-1*, bacteroids undergo premature degradation (McAdam et al., 2018). Together, these data strongly indicate that aging of symbiotic nodules is accompanied by a decrease in the level of GAs, which suggests involvement of GAs in the delay of nodule senescence (Figure 10A,B).

No significant differences in *PsGA20ox1* and *PsGA2ox1* transcript abundance were seen during nodule aging of the mutant SGEFix^-^-2 (*sym33*), blocked at the earliest stage of symbiosis. In addition, a low level of GA_3_ labeling in nodules of the mutant SGEFix^-^-2 (*sym33*) was observed. Recently, we have shown early activation of senescence-associated genes in this mutant at 2 WAI (Serova et al., 2018). This activation may explain the low level of GAs in the mutant. The low level of bioactive GAs in the mutant SGEFix^-^-2 (*sym33*) is accompanied by the absence of infected cells due to the absence of bacterial release (Tsyganov et al., 1998). These results may reflect the necessity of GAs for bacterial release (Tatsukami and Ueda, 2016) and infected cell development.

Analysis of the mutant SGEFix^-^-1 (*sym40*), blocked at a later stage of symbiosis than mutant SGEFix^-^-2 (*sym33*) (Tsyganov et al., 2011) and manifesting signs of premature nodule senescence (Tsyganov et al., 1998; Serova et al., 2018), demonstrated a similar transcriptional pattern of *PsGA20ox1* to that observed in wild-type nodules and a significant increase in *PsGA2ox1* mRNA as well as low GA_3_ label content in 4-week-old nodules. These data are suggestive of a negative role of GAs during nodule senescence.

Aging of the symbiotic nodule is regulated by changes in gene expression. In particular, the expression levels of genes encoding cysteine proteases, transcription factors, enzymes of GA deactivation, and enzymes of ethylene and ABA biosynthesis were shown to increase during senescence of *M. truncatula* and pea nodules (Kardailsky and Brewin, 1996; Van de Velde et al., 2006; D’haeseleer et al., 2010; de Zélicourt et al., 2012; Karmarkar, 2014; Serova et al., 2017, 2018). To assess the effect of GAs on the senescence of the pea symbiotic nodules, we analyzed the mRNA levels of senescence-associated genes in 2-, 4-, and 6-week-old nodules of wild-type plants treated with exogenous GA_3_ relative to untreated plants. A less pronounced increase in transcript levels of genes *PsCyp1*, *PsCyp1*5, and *PsTPP* was shown during aging of the nodules of GA_3_-treated plants, in contrast to the nodules of the untreated plants. In addition, the transcript abundance of all analyzed genes was significantly higher in the nodules of untreated plants than in nodules of plants treated with GA_3_ at 6 WAI, which may indicate a delay of nodule senescence upon treatment with exogenous GA_3_ (Figure 10C). It is known that cysteine proteases carry out large-scale protein degradation during nodule senescence (Pladys et al., 1991; Granell et al., 1992; Kardailsky and Brewin, 1996; Van de Velde et al., 2006; Pérez Guerra et al., 2010). Thus, our data suggest that a decrease in GAs is required to induce the degradation processes during nodule aging. Previously, it was shown that the expression level of the *ATB2* gene was up-regulated during senescence of *M. truncatula* (D’haeseleer et al., 2010) and pea (Serova et al., 2017) nodules. The transcript level of *PsATB2* was reduced in the nodules of GA_3_-treated plants in comparison with the nodules of untreated plants (Figure 10C). This may indicate possible regulation of nodule senescence by GAs through an effect on the bZIP transcription factor. Similar expression patterns were observed for genes encoding key enzymes of ethylene biosynthesis, ACC synthase (*PsACS2*), ACC oxidase (*PsACO1*), and aldehyde oxidase (*PsAO3*), an enzyme catalyzing the last step of ABA biosynthesis (Figure 10C). It is known that ethylene and ABA promote nodule senescence (Swamy and Smith, 1999; Guinel, 2015; Serova et al., 2017, 2018). In addition, elevation of ethylene levels in the *na-1* mutant was previously demonstrated (Ferguson et al., 2011). Thus, a lower expression level of *PsACS2*, *PsACO1*, and *PsAO3* genes in the nodules of GA_3_-treated plants may also suggest integration of hormonal signaling during nodule senescence. During the nodule aging of both GA_3_-treated and untreated plants, down-regulation of one GA biosynthetic gene, *PsGA20ox1*, and up-regulation of one GA deactivation gene, *PsGA2ox1*, were demonstrated (Figure 10C). However, only *PsGA2ox1* transcript abundance was significantly lower in the nodules of GA_3_-treated plants than in those of untreated plants at 6 WAI. Thus, it can be assumed that regulation of bioactive GAs in the nodules of GA_3_-treated plants occurred mainly by regulation of GA biosynthesis.

Two-week-old nodules of GA_3_-treated plants were about two times larger than the nodules of untreated plants, probably due to involvement of GAs in cell division and cell elongation (Hedden and Thomas, 2012). The number of nodules formed by GA_3_-treated plants was nearly two times smaller than that in untreated plants. Previously, a similar concentration (10^-6^ M) of GA_3_ had no effect on nodule number in the wild type, but a higher concentration (10^-3^ M) inhibited nodule formation (Ferguson et al., 2005). This contradiction may be due to differences in plant genotypes and experimental conditions. However, GA_3_ treatment of the severely inhibited GA mutant *na-1*, characterized as having a low number of nodules, restored nodule number, which suggests a direct role for GAs in nodule development (Ferguson et al., 2005). Most of the GA mutants were characterized by a reduced number of nodules, but increased nodule dry weight. It has been suggested by Ferguson et al. (2005) that there is a compensation mechanism to regulate the size of individual nodules, depending on the number of nodules per plant.

Pronounced meristem bifurcation was observed in 4- and especially 6-week-old nodules of GA_3_-treated plants (Figure 10C). This may indicate the involvement of GAs in functioning of the nodule meristem. Previously, it was shown that meristem bifurcation during root branching is under phytohormonal control, including auxin, cytokinins, and ethylene (Gola, 2014). GA_3_ immunolocalization in pea nodules performed in this study and previous studies carried out on Arabidopsis and pea plants (Ferguson et al., 2005, 2011; Achard et al., 2009) confirms the involvement of GAs in nodule meristem functioning. It should be noted that 6-week-old nodules of GA_3_-treated plants also had a senescence zone, but it occupied a smaller part (about 34%) of the nodule than in nodules of the untreated plants (about 62%). This may be due to the meristem bifurcation observed in the nodules of GA_3_-treated plants. It is known that senescence of the nodule is associated with arrested division of meristem cells and the discontinuance of rhizobial release from infection droplets (Guinel, 2015).

The main part of mature nodules of GA_3_-treated plants was represented by the nitrogen fixation zone (Figure 10C). Also, the high content of GA_3_ label in cells of the nitrogen fixation zone in young and mature pea nodules indicates a possible involvement of GAs in the functioning of nitrogen fixation nodules and, consequently, delay of nodule senescence. In *A. thaliana*, it was shown that DELLAs repress the inhibitory effect of JASMONATE ZIM-domain proteins (JAZ), which are transcriptional regulators, on the expression of jasmonate (JA) responsive genes, such as lipoxygenase and defense genes (Hou et al., 2010). On the contrary, GAs suppress the expression of JA-responsive genes via DELLA degradation. Thus, the involvement of GAs in suppressing the response to JA-induced signaling may be one of the mechanisms by which GAs contribute to the delay of nodule senescence, which is regarded as a delayed response of the plant to rhizobia as a potential pathogen (Mellor, 1989). Furthermore, an up-regulation of transcripts of JA biosynthesis genes was observed during *M. truncatula* nodule senescence (Van de Velde et al., 2006).

## Conclusion

In this study, the involvement of bioactive GAs in nodule senescence of pea wild-type and nodule development mutants was studied by assessing transcriptional patterns of GA metabolism genes, and through GA_3_ immunolocalization and pharmacological analyses. A decrease in GA content during nodule aging was demonstrated at the transcriptional level via a down-regulation of the GA biosynthesis gene, *PsGA20ox1*, and an up-regulation of the GA deactivation gene, *PsGA2ox1*, and also by the immunolocalization of bioactive GA_3_ in the mutant and wild-type nodules. These results indicate a role of GAs in a delay of nodule senescence. A down-regulation of senescence-associated genes, a decrease of the senescence zone, and an increase of the nitrogen fixation zone in nodules of wild-type plants treated with exogenous GA_3_ confirm a negative regulation of nodule senescence by GAs and involvement of GAs in the functioning of the mature nodule.

## Data Availability

All datasets generated for this study are included in the manuscript and/or the Supplementary Files.

## Author Contributions

IT designed the experiments. TS and AT performed the experiments. TS and VT analyzed the data and wrote the manuscript.

## Conflict of Interest Statement

The authors declare that the research was conducted in the absence of any commercial or financial relationships that could be construed as a potential conflict of interest.
