# Supplementary material for: Gibberellins Inhibit Nodule Senescence and Stimulate Nodule Meristem Bifurcation in Pea (Pisum sativum L.)
Source: Front Plant Sci. 2019 Mar 15;10:285. doi: 10.3389/fpls.2019.00285 (PMC6428903; doi:10.3389/fpls.2019.00285)
Supplement: Supplementary file 1 [file Table_1.docx]

Supplementary Material

Gibberellins Inhibit Nodule Senescence and Stimulate Nodule Meristem Bifurcation in Pea (*Pisum sativum* L.)

Tatiana A. Serova^1^, Anna V. Tsyganova^1^, Igor A. Tikhonovich^1,2^, Viktor E. Tsyganov^1^*

^1^Laboratory of Molecular and Cellular Biology, Department of Biotechnology, All-Russia Research Institute for Agricultural Microbiology, Pushkin 8, Saint Petersburg, Russia

^2^Saint-Petersburg State University, Department of Genetics and Biotechnology, Saint Petersburg, Russia

*** Correspondence:**Viktor E. Tsyganov
tsyganov@arriam.spb.ru


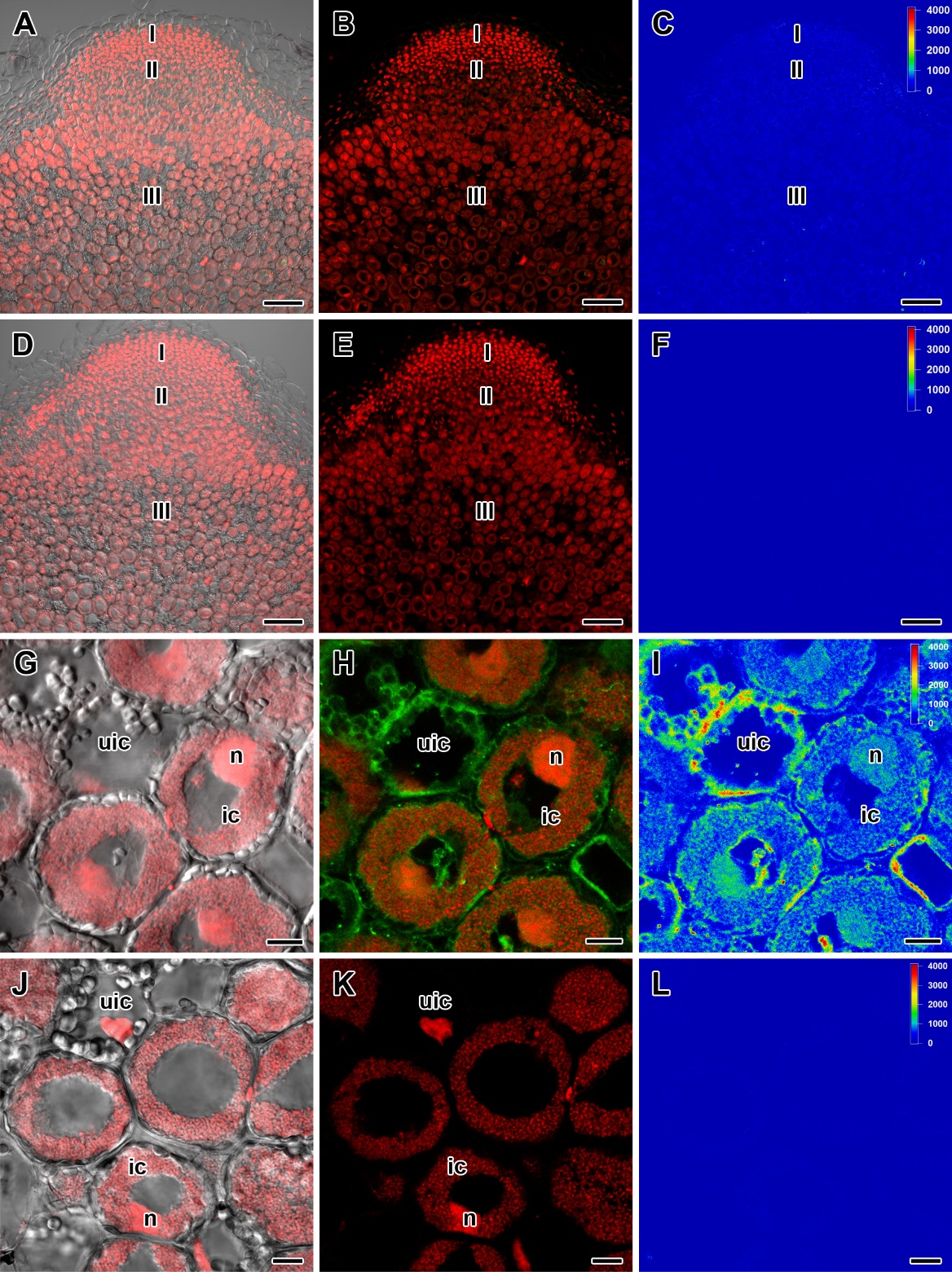


**Supplementary Figure 1.** The controls show specificity of antibodies to gibberellin (GA_3_). To confirm specificity of the anti-GA_3_ antibody, slices of wild-type SGE 2-week-old nodules were incubated with GA_3_-specific antibodies supplemented with GA_3_-BSA conjugate before immunostaining (**A–C**). Note the absence in fluorescence in the whole nodule. Primary anti-GA_3_ antibodies were omitted as a negative control, resulting in the absence of fluorescence (**D–F, J–L**). The controls show specificity of antibodies to gibberellin (GA_3_) in nuclei (**G–L**). A differential interference contrast microscopy image merged with laser scanning confocal microscopy image in red channel **(A, D, G, J).**  Merged images of laser scanning confocal microscopy in green and red channels (**B, E, H, K**). Heat map provides a color code of fluorescence signal intensities (**C, F, I, L**). GA_3_ in green, nuclei and bacteria in red. Scale bar = 100 µm (**A–F**), 10 µm (**G–L**).

**
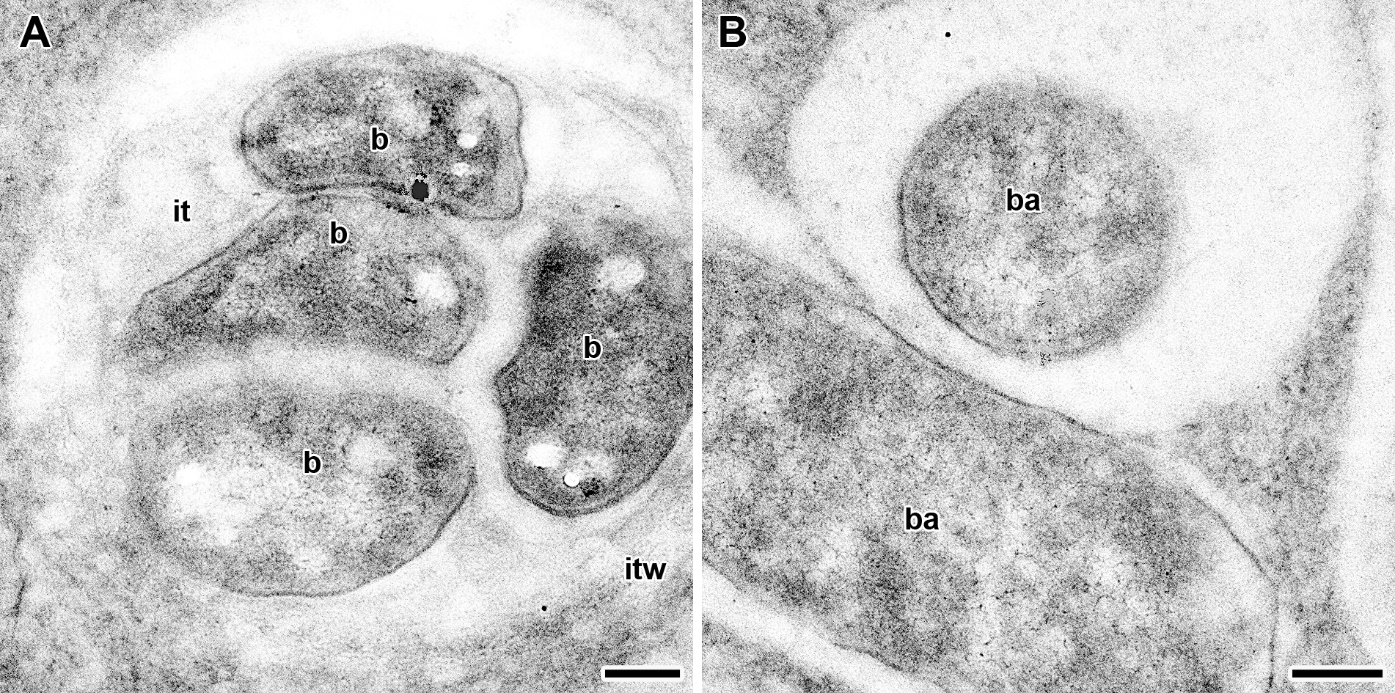
**

**Supplementary Figure 2.** Transmission electron micrographs of cells from nodule of wild-type SGE at 2 weeks after inoculation treated as negative control to GA_3_ immunogold labeling. Gold particles were absent when cells were treated after the omission of the primary antibody (**A**), with unspecific secondary antibody (**B**). It was used secondary goat anti-mouse IgG MAb conjugated to 10 nm diameter colloidal gold. it, infection thread; itw, infection thread wall; b, bacterium; ba, bacteroid. (**A**) Infection thread, (**B**) Mature bacteroids. Scale bar = 200 nm.

**
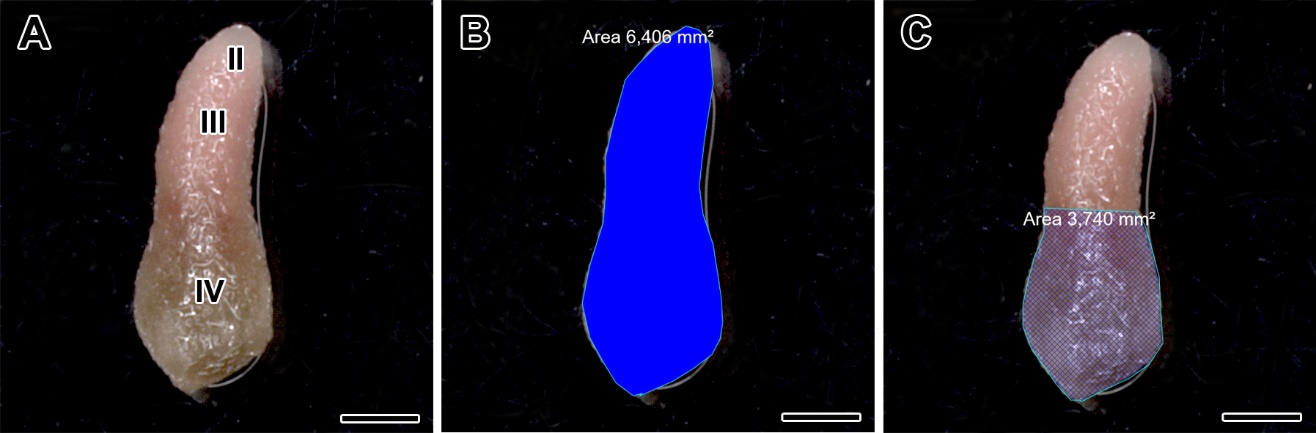
**

**Supplementary Figure 3.** Selection and measurement of area of wild-type nodule at 6 weeks after inoculation. General view of nodule **(A)**, selection and measurement of whole nodule area (blue background) **(B)**, selection and measurement of area of senescence zone in nodule (blue mesh background) **(C)**. Areas were selected and measured with AxioVision Rel. 4.8 software (Carl Zeiss). Zones of nodule are designated by Roman numerals: II – infection zone, III – fixation zone, IV – senescence zone. Scale bar = 1 mm.

**
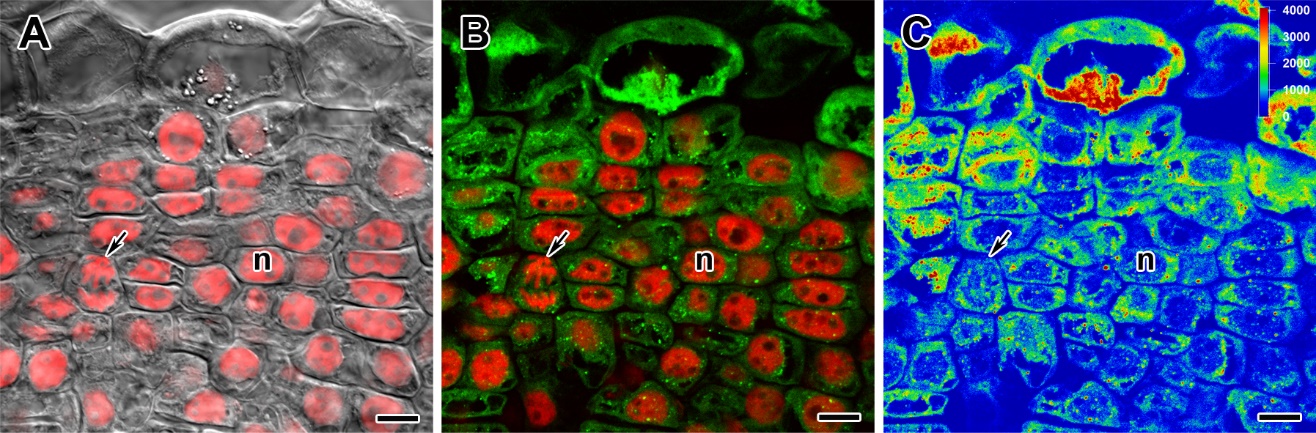
**

**Supplementary Figure 4.** Immunolocalization of gibberellin (GA_3_) in meristem of nodules of wild-type SGE at 2 weeks after inoculation. n, nucleus. Arrow indicates mitosis. A differential interference contrast microscopy image merged with laser scanning confocal microscopy image in red channel **(A).** Merged images of laser scanning confocal microscopy in green and red channels **(B)**. Heat map provides color code of fluorescence signal intensities **(C)**. Visualization of GA_3_ by the Alexa Fluor 488 conjugated secondary antibody (green), nuclei and bacteria stained with propidium iodide (red). Scale bar = 10 µm.

**
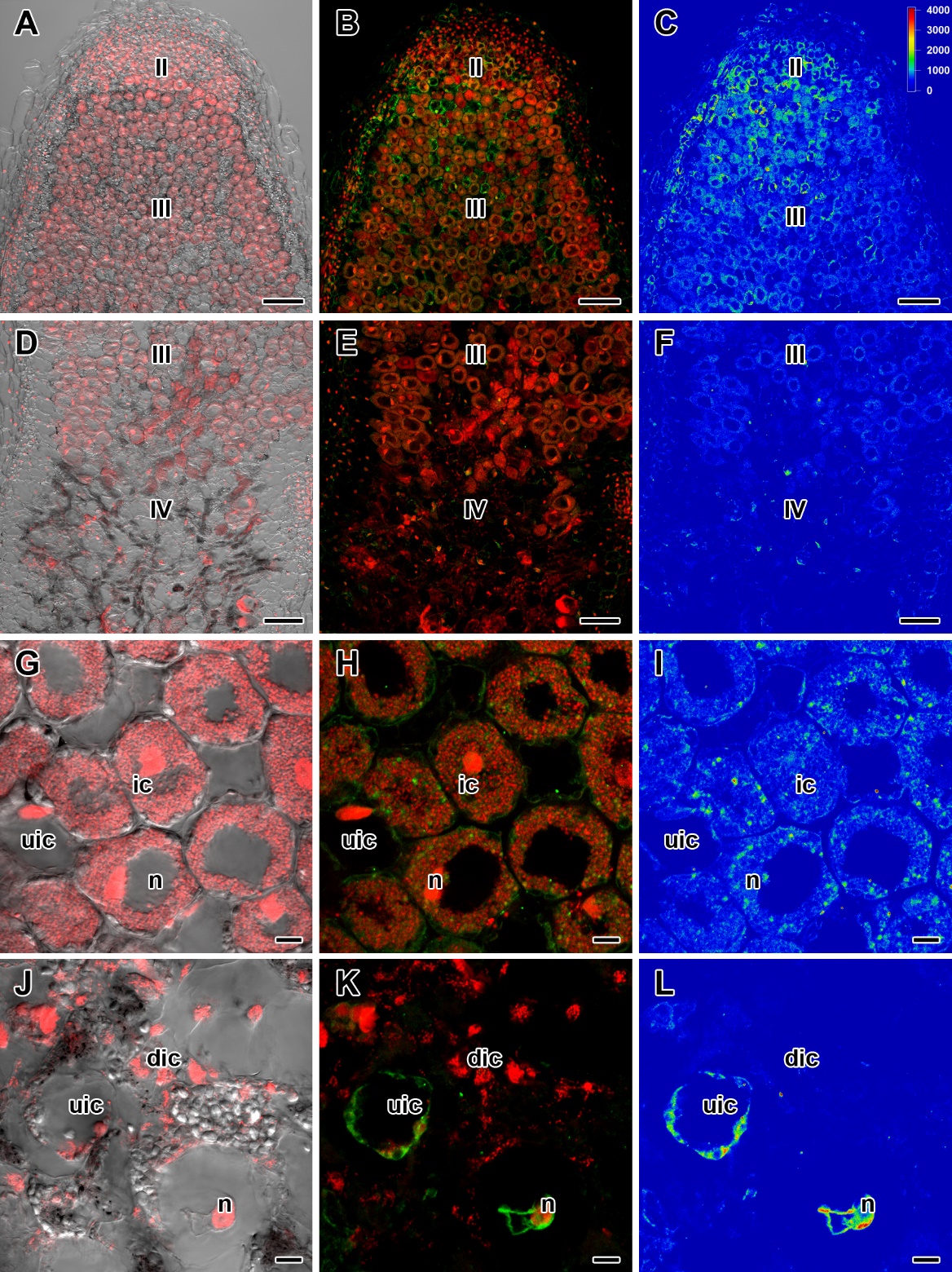
**

**Supplementary Figure 5.** Immunolocalization of gibberellin (GA_3_) in nodules of wild-type plants **(A–F)** and cells in central part of nodules **(G–L)** at 6 weeks after inoculation. Zones of nodule are designated by Roman numerals: II – infection zone, III – fixation zone, IV – senescence zone. ic, infected cell; dic, degrading infected cell; uic, uninfected cell; n, nucleus. A differential interference contrast microscopy image merged with laser scanning confocal microscopy image in red channel **(A, D, G, J).**  Merged images of laser scanning confocal microscopy in green and red channels (**B, E, H, K**). Heat map provides color code of fluorescence signal intensities **(C, F, I, L)**. Visualization of GA by the Alexa Fluor 488 conjugated secondary antibody (green), nuclei and bacteria stained with propidium iodide (red). Scale bar (**A-F**) = 100 µm, (**G-L**) = 10 µm.

**
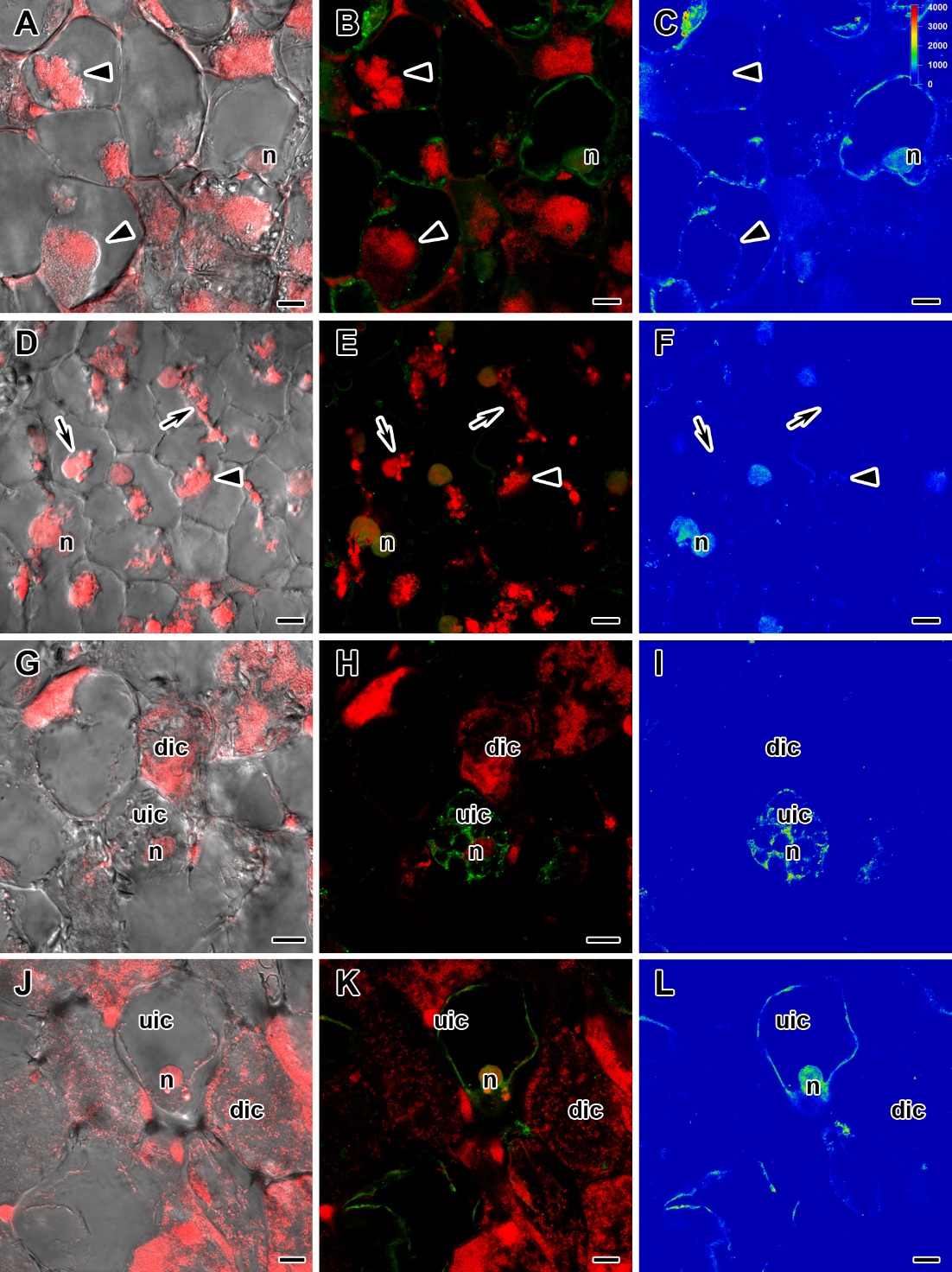
**

**Supplementary Figure 6.** Immunolocalization of gibberellin (GA_3_) in cells in central part of nodules of pea mutants (SGEFix^−^-1 (*sym40*) **(A–C)**, SGEFix^−^-2 (*sym33*) **(D–F)**, SGEFix^−^-3 (*sym26*) **(G–I)** and SGEFix^−^-7 (*sym27*) **(J–L)**) at 4 weeks after inoculation. dic, degrading infected cell; uic, uninfected cell; n, nucleus. Arrow indicates infection thread, arrowhead indicates infection droplet. A differential interference contrast microscopy image merged with laser scanning confocal microscopy image in red channel **(A, D, G, J).**  Merged images of laser scanning confocal microscopy in green and red channels (**B, E, H, K**). Heat map provides color code of fluorescence signal intensities **(C, F, I, L)**. Visualization of GA by the Alexa Fluor 488 conjugated secondary antibody (green), nuclei and bacteria stained with propidium iodide (red). Scale bar = 10 µm.
